# Impaired 53BP1/RIF1 DSB mediated end-protection stimulates CtIP-dependent end resection and switches the repair to PARP1-dependent end joining in G1

**DOI:** 10.18632/oncotarget.11023

**Published:** 2016-08-02

**Authors:** Ali Bakr, Sabrina Köcher, Jennifer Volquardsen, Cordula Petersen, Kerstin Borgmann, Ekkehard Dikomey, Kai Rothkamm, Wael Y. Mansour

**Affiliations:** ^1^ Laboratory of Radiobiology & Experimental Radiooncology, University Medical Center Hamburg–Eppendorf, Hamburg, Germany; ^2^ Department of Radiotherapy and Radiooncology, University Medical Center Hamburg–Eppendorf, Hamburg, Germany; ^3^ Tumor Biology Department, National Cancer Institute, Cairo University, Egypt

**Keywords:** double strand break ends processing, 53BP1, RIF1, ATM, A-EJ

## Abstract

End processing at DNA double strand breaks (DSB) is a decisive step in repair pathway selection. Here, we investigated the role of 53BP1/RIF1 in limiting BRCA1/CtIP-mediated end resection to control DSB repair pathway choice. ATM orchestrates this process through 53BP1 phosphorylation to promote RIF1 recruitment. As cells enter S/G2-phase, end resection is activated, which displaces pATM from DSB sites and diminishes 53BP1 phosphorylation and RIF1 recruitment. Consistently, the kinetics of ATM and 53BP1 phosphorylation in S/G2-phase concur. We show that defective 53BP1/RIF1-mediated DSB end-protection in G1-phase stimulates CtIP/MRE11-dependent end-resection, which requires Polo-like kinase 3. This end resection activity in G1 was shown to produce only short tracks of ssDNA overhangs, as evidenced by the findings that in 53BP1 depleted cells, (i) RPA focus intensity was significantly lower in G1 compared to that in S/G2 phase, and (ii) EXO1 knockdown did not alter either number or intensity of RPA foci in G1 but significantly decreased the RPA focus intensity in S/G2 phase. Importantly, we report that the observed DSB end resection in G1 phase inhibits DNA-PK-dependent nonhomologous end joining but is not sufficient to stimulate HR. Instead, it switches the repair to the alternative PARP1-dependent end joining pathway.

## INTRODUCTION

Double strand breaks (DSBs) are dangerous chromosomal lesions. Failure to accurately repair DSBs can lead to gross chromosomal rearrangements or mutations at the break site, which can cause cell death, cell transformation, and tumorigenesis [1]. Two mechanistically distinct pathways have evolved to eliminate DSBs from the genome: non-homologous end joining (NHEJ) and homologous recombination (HR). NHEJ is basically active throughout the cell cycle but preferable in G1 phase. NHEJ operates independently of DNA sequence around the break and ensures that DSB ends are held in proximity to permit their direct ligation [2, 3]. HR is an error-free mechanism as it uses the sister chromatid as a template. Accordingly, HR is active only during the S and G2 cell cycle phases [4]. Molecular regulation of DSB repair pathway choice has been the subject of intense study for quite some time in many labs, including our own. Previously we have reported a functional hierarchy between the main DSB repair pathways and their alternative back-up mechanisms [5]. This hierarchy is regulated by the NHEJ heterodimer KU70/80, which protects DSB ends from end resection and hence ensures they are repaired *via* accurate NHEJ. Consequently, in KU-deficient cells, DSB repair is switched not only to HR but mainly also to an inaccurate alternative end joining (Alt-EJ) mechanism called PARP1-dependent end joining (PARP1-EJ) [6, 7]. This hierarchy is regulated by many factors, including the initial processing of the DSB ends [8]. DNA processing is primarily regulated in a cell cycle dependent manner. In G1 phase, DSB ends are protected and NHEJ is utilized as the main repair pathway. However, as cells enter S and G2 phase, 5′ end resection is activated to produce DSBs with 3′ single stranded overhangs to commit the repair to HR [8, 9]. Breast cancer 1 (BRCA1) and C-terminal binding protein-interacting protein (CtIP) are known to be critical factors for the DSB end resection process. CtIP nuclease activity is accelerated by the binding to BRCA1. CtIP is phosphorylated by cyclin-dependent kinase (CDK) at the G1-S transition to initiate end resection and commit the repair to HR [10, 11]. Consistently, the end resection process can be inhibited by arresting cells in G1 phase or by inhibiting CDK activity [6, 10, 11]. This finding suggests a straightforward model in which a phosphorylation-dependent switch at the G1-S transition turns on the resection process. However, further investigations have revealed much greater complexity in the determination of DSB repair pathway choice. We and others have previously reported an antagonistic relationship between BRCA1 and the DNA damage response (DDR) protein p53-binding protein 1 (53BP1). 53BP1 suppresses HR and is a positive regulator of NHEJ by protecting DSB from BRCA1-mediated end processing [12–14]. Therefore, the physical presence of 53BP1 at DSB ends is required for its HR-suppressive activity. Indeed, several studies have shed light on the involvement of other factors that work together with 53BP1 in protecting DSB ends. Among these factors, RAP1-interacting factor 1 (RIF1) and Pax transactivation domain-interacting protein (PTIP) were shown to be recruited to DSB sites in a 53BP1-dependent manner [15–20]. This suggests that 53BP1 acts as a scaffold protein to facilitate the recruitment of the end protection factors RIF1 and PTIP to the DSB site and hence committing the repair to NHEJ. Interestingly, the recruitment of RIF1 and PTIP was found to depend on the ataxia telangiectasia mutated (ATM) -mediated 53BP1 phosphorylation [15, 18, 21], putting ATM at the center of the end protection process. This raises a paradox because it is already known that ATM is critical for the end resection process and that ATM-deficient cells are deficient in end resection [22–30].

In the current study, we show that ATM-dependent 53BP1 phosphorylation is more pronounced in G1 than in S/G2 phase. We further find that the differential 53BP1 phosphorylation in different cell cycle phases results from a different binding pattern of phosphorylated ATM (pATM) to DSB in different cell cycle phases. While intense pATM foci are formed in G1 phase, pATM foci are less intense in S/G2, indicating that more pATM molecules are bound to DSB in G1 phase. Interestingly, defective end protection in G1 stimulates CtIP- and MRE11- but not EXO1-dependent end resection. This short track DSB end resection inhibits NHEJ but fails to activate HR. Importantly; we report here for the first time that in the absence of 53BP1/RIF1, the resected ends in G1 cells are processed by alternative PARP1-EJ.

## RESULTS

### 53BP1 and RIF1 counteract BRCA1/CtIP-mediated end resection to regulate DSB repair pathway choice

**Figure 1:**
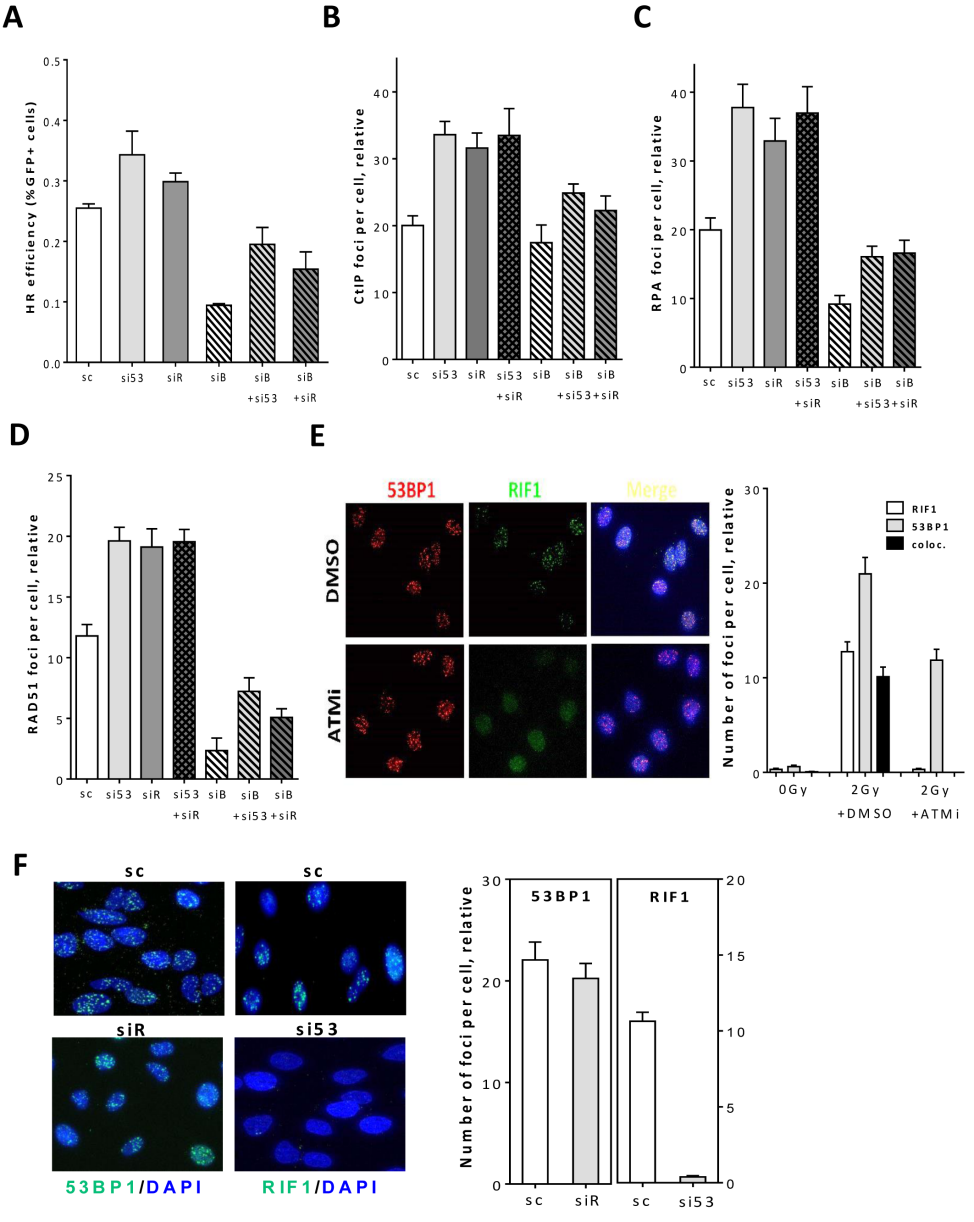
53BP1/RIF1 work epistatically to counteract BRCA1/CtIP-mediated end resection to regulate DSB repair pathway choice **A.** HeLa cells harboring pGC were treated with the indicated siRNAs before transfection with I-SceI-expressing vector to induce DSBs. After 48h, the percentage of GFP^+^ cells was measured as an indication for HR efficiency. **B.**-**D.** Asynchronous A549 cells were treated with the indicated siRNAs before irradiation with 2Gy and CtIP **B.** and RPA foci **C.** were monitored at 2h while RAD51 **D.** foci were enumerated at 4h. **E.** Left panel: representative photos for the colocalization between 53BP1 and RIF1 foci in A549 cells at 2h after 2Gy. Right panel: quantitation of 53BP1, RIF1 or colocalized foci in the absence (DMSO) or presence of ATM inhibitor (ATMi). **F.** Left panel: representative micrographs for 53BP1 or RIF1 foci in irradiated cells after depletion of either proteins. Right panel: quantitation of experiments presented in the left panel. At least 100 nuclei were counted. In all cases, the number of foci measured in non-irradiated cells was subtracted (relative). Shown are the mean ±SEM for three independent experiments. sc: scrambled RNA, si53: si53BP1, siR: siRIF1, siB: siBRCA1, and siC: siCtIP.

Several studies including our own have previously reported that the DDR proteins 53BP1 and RIF1 limit the resection of DSB ends, therefore inhibiting HR and committing the repair to NHEJ [12–14, 20, 21]. Firstly, we sought to recapitulate these data and therefore, we analyzed the effect of RIF1 or 53BP1 downregulation (Figure S1) on HR and NHEJ repair pathways in HeLa cells harboring stably integrated copies of pGC or pEJ reporter plasmids, respectively. As anticipated, depletion of either 53BP1 or RIF1 enhanced HR but inhibited NHEJ (Figure 1A) [12] and data not shown. Knockdown of the end resection factor BRCA1 compromised HR. This anti-HR role of 53BP1 and RIF1 was attributed to their function in limiting end resection as evidenced by increased number of CtIP, RPA and RAD51 foci upon 53BP1 or RIF1 depletion (Figure 1B-1D). Importantly, double knockdown of RIF1 and 53BP1 (Figure S1) was not dissimilar from single knockdown regarding the number of CtIP, RPA, or RAD51 (Figure 1B-1D), indicating an epistatic relationship of 53BP1 and RIF1 in DSB end protection [17, 19, 21]. Indeed, both proteins colocalized after IR and as expected inhibition of ATM suppressed this colocalization (Figure 1E). Furthermore, while 53BP1 focus formation was not affected by RIF1 depletion, RIF1 did not form foci upon 53BP1 knockdown (Figure 1F). Collectively, these data suggest that RIF1 is a downstream co-factor of ATM/53BP1 in protecting DSB ends from end resection processes.

Similar to what we previously reported for 53BP1 [12], RIF1 forms more intense foci in G1 compared to S/G2 phase (Figure S2A). Importantly, RIF1 foci intensities in S/G2 cells were found to be antagonized by the end resection factors BRCA1 and CtIP, as evidenced by an increase in RIF1 foci intensity in S/G2 phase upon knockdown of either protein, thus being comparable to that in G1-phase (Figure S2A). On the other hand, RIF1 depletion led to a 2-fold and 1.6-fold increase in BRCA1 and CtIP IRIF numbers in S/G2 cells, respectively (Figure 1B & S2B), confirming its role in regulating DSB repair pathway choice. Together, these data demonstrate that the end protection factors 53BP1-RIF1 crosstalk with the end resection factors BRCA1-CtIP to regulate DSB end processing and repair pathway choice.

BRCA1-deficient cells are sensitive to PARP inhibitor olaparib and mitomycin-C (MMC) due to impaired end resection and hence HR deficiency. Previously, it was shown by many labs, including our own, that 53BP1 depletion rescues survival of BRCA1-deficient cells after olaparib or MMC treatment [12–14, 19, 20, 31]. Consistently, additional knockdown of RIF1 or 53BP1 significantly increased HR efficiency in BRCA1-depleted cells as measured by the HR repair substrate pGC (Figure 1A & S1). Expectedly, numbers of CtIP, RPA and RAD51 foci in BRCA1-depleted cells were significantly restored after additional knockdown of RIF1 similar to that after 53BP1 knockdown (Figure 1B-1D & S1). This rescue effect was further recapitulated in BRCA1-deficient HCC1937 cells (Figure S2C & S2D). In conclusion, this data shows that both 53BP1 and RIF1 work epistatically to counteract BRCA1/CtIP-mediated DSB-end resection to control repair pathway choice.

### End resection displaces ATM from DSBs in S/G2 phase to initiate HR

**Figure 2:**
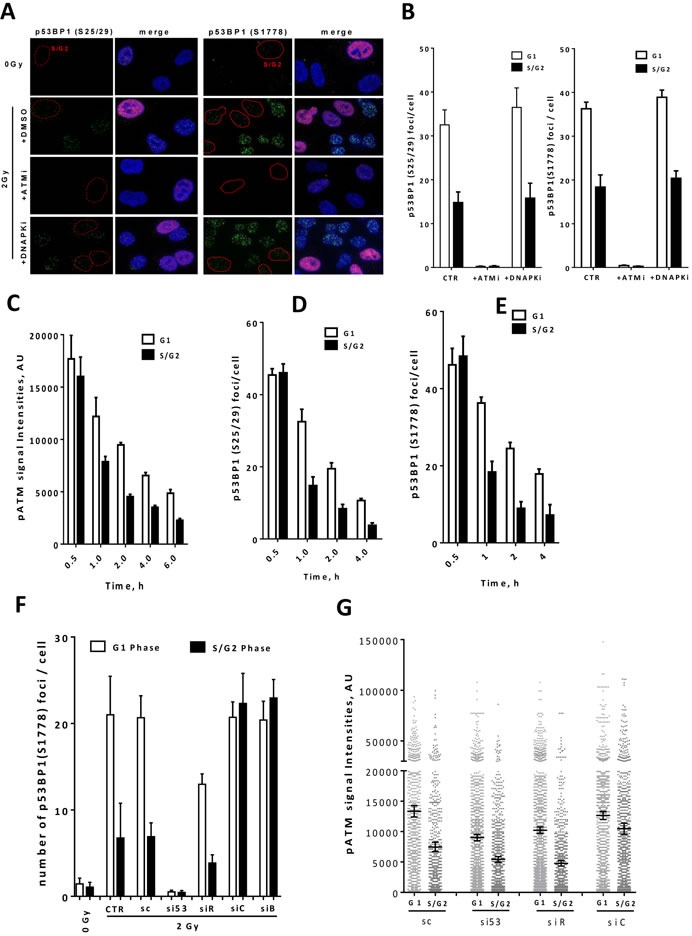
ATM regulates 53BP1-RIF1-mediated end protection by phosphorylating 53BP1 in a cell cycle dependent manner **A.** Representative micrographs for p53BP1 (S25/29; left panel & S1778; right panel) foci in asynchronous A549 control cells (DMSO) or after inhibition of either ATM (ATMi) or DNA-PK (DNA-PKi). Nuclei were counterstained with CenpF to distinguish G1 (CenpF^−^) from S/G2 (CenpF^+^) cells. **B.** Quantitation of the experiments presented in ‘A’. About 100 nuclei were counted. **C.** Focus intensity of pATM in G1 and S/G2 cells at the indicated time points after 2Gy. The intensities of about 2000 foci were quantified in each cell cycle phase. **D.**-**E.** Foci number of p53BP1 at **D.** S25/29 or **E.** S1778 in G1 *vs* S/G2 cells at the indicated time points after 2Gy. **F.** A549 cells were treated with the indicated siRNAs and p53BP1 (S1778) foci were enumerated at 2h post-2Gy in G1 and S/G2 phases. **G.** pATM focus intensity of about 2000 foci was measured at 1h post-2Gy in G1 and S/G2 A549 cells treated as in ‘F’. Data presented as the mean ±SEM of two (for p53BP1 foci) or three (for pATM focus intensity) independent experiments.

It was previously shown that the repair function of 53BP1 requires both its localization to DSBs and its phosphorylation by ATM in response to DNA damage [32]. Since ATM regulates the recruitment of the end protection factor RIF1 through phosphorylation of its upstream cofactor 53BP1 ([16, 21] and Figure 1E), we sought to analyze the level of ATM-dependent 53BP1 phosphorylation throughout the cell cycle. In response to IR, ATM phosphorylates 53BP1 at several serine residues including 25, 29 and 1778. Interestingly, immunostaining analysis revealed different patterns of 53BP1 phosphorylation (p53BP1) at these residues in different cell cycle phases (Figure 2A). The signals of p53BP1 at S25/29 and S1778 residues were half as intense in S/G2 compared to those in G1 cells (Figure 2A & 2B). As anticipated, ATM but not DNA-PK inhibition diminished the phosphorylation of 53BP1 at the aforementioned sites (Figure 2B). These data suggest that DNA damage-induced 53BP1 phosphorylation is ATM-mediated and cell cycle-dependent. Since ATM is equally activated throughout all cell cycle phases, we hypothesized that activated ATM (i.e. phosphorylated at S1981) is more enriched at DSB sites in G1 than in S/G2, which would explain the different patterns of 53BP1 phosphorylation in different cell cycle phases. To verify this hypothesis, we measured the kinetics of pATM (S1981) foci intensity in unsynchronized A549 cells after 2Gy in different cell cycle phases using the S/G2 marker CenpF. At 30 min, pATM foci intensity was comparable in G1 and S/G2 phases. However, it decreased faster with time in S/G2 phase compared to G1 (Figure 2C), indicating faster displacement of active ATM from DSB sites in S/G2 phase. Interestingly, p53BP1 foci number showed concurrent kinetics to that of pATM, with comparable foci numbers in G1 and S/G2 phases at 30 min and subsequent faster decrease in foci number in S/G2 compared to that in G1 phase (Figure 2D & 2E). We confirmed the specificity of the pATM and p53BP1 antibodies used for these experiments (Figure S3A & S3B). The observed decrease in pATM foci intensity and p53BP1 foci number in S/G2 phase probably indicates an ongoing DSB end resection. In order to verify this, we measured foci intensity of pATM and number of p53BP1 foci after depletion of BRCA1 or CtIP using siRNA. While depletion of BRCA1 or CtIP did not affect either pATM foci intensity or p53BP1 foci number in G1 phase, it significantly increased both of them in S/G2 cells (Figure 2F & 2G), indicating that end resection is responsible for the fast decline in the intensity of pATM and the number of p53BP1 foci in S/G2 phase.

Taken together, this indicates therefore that the activation of end resection in S/G2 displaces activated ATM (i.e. pATM) at the DSB site, decreases 53BP1 phosphorylation and RIF1 recruitment and hence commits the repair to HR.

### Depletion of 53BP1 or RIF1 leads to accumulation of BRCA1, CtIP, RPA but not RAD51 foci in G1 cells

**Figure 3:**
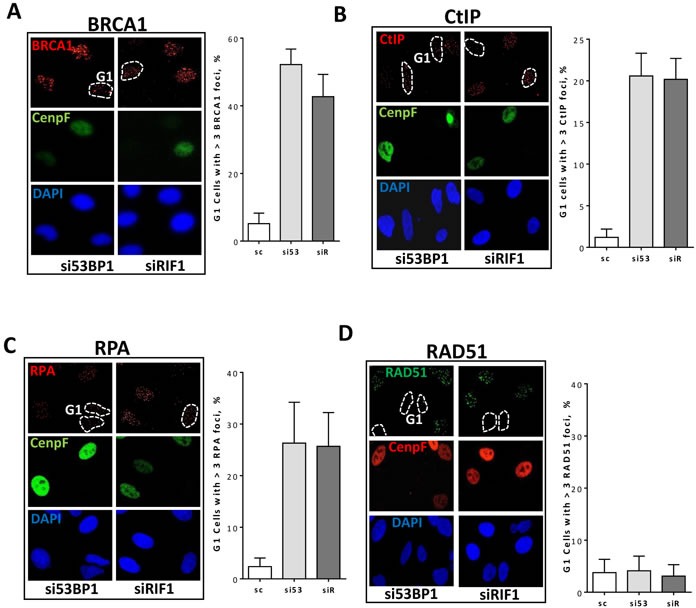
53BP1-RIF1 loss enhances resection of DSBs but is not enough to drive HR in G1 cells Asynchronous A549 cells were treated with the indicated siRNA for 48h and irradiated with 2Gy. Cells were then fixed and immunostained for BRCA1, CtIP, and RPA after 2h or for RAD51 after 4h post irradiation. Nuclei were counterstained with CenpF to distinguish G1 and S/G2 cells. Shown are the percentages of G1 (CenpF^−^) cells with more than three BRCA1 **A.**, CtIP **B.**, RPA **C.**, and RAD51 **D.** foci.

Interestingly, depletion of either 53BP1 or RIF1 decreases pATM foci intensity in G1 cells (Figure 2G), which may indicate an activated end resection in G1 phase. To verify this, we sought to monitor BRCA1, CtIP, and RPA foci formation in 53BP1 or RIF1 depleted G1 cells. Intriguingly, after depletion of RIF1 or 53BP1, we observed significant (*P < 0.001)* increases in the formation of BRCA1 (Figure 3A & Figure S4A-S4C), CtIP (Figure 3B & Figure S4D-S4F), and RPA (Figure 3C & Figure S4G-S4I) foci in G1-cells, indicating that DSB ends are resected in G1 after suppression of 53BP1-RIF1 mediated end protection. Importantly, 53BP1- or RIF1-depleted cells failed to recruit RAD51 to the resected DSB ends in G1 (Figure 3D & Figure S4J-S4L), suggesting the presence of an additional mechanism that negatively controls HR in G1 phase.

Of note, adding the dNTP analog EdU post-2Gy (i.e. 15 min before the fixation) to detect cells in early S-phase with undetectable CenpF staining showed a consistency in detecting G1-cells based on negative CenpF staining (Figure S5).

### Resection of DSB ends in 53BP1/RIF1-depeted cells in G1-phase is MRE11- and CtIP- but not EXO1-dependent

**Figure 4:**
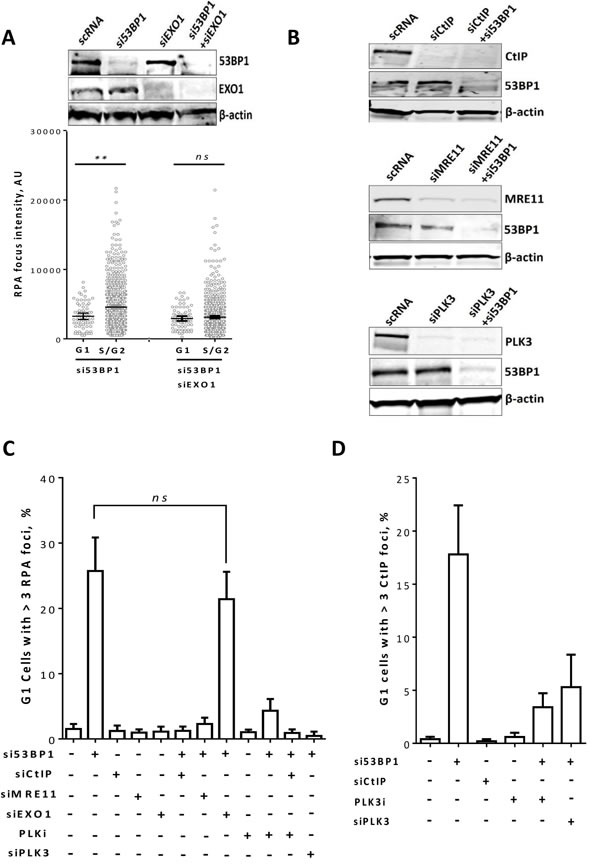
CtIP and MRE11 but not EXO1 are required for end resection in 53BP1-depleted G1 cells **A.** A549 cells were treated as in Figure 4 with the indicated siRNAs and efficient knockdown of 53BP1 and/or EXO1 was confirmed by Western blot (upper panel). Lower panel: The focus intensity of RPA was measured in 53BP1- and/or EXO1- depleted cells at 2h post-2Gy in G1 and S/G2. **B.** Immunoblots showing efficient siRNA-mediated knockdown of CtIP and/or 53BP1 (upper panel), MRE11 and/or 53BP1 (middle panel), or PLK3 and/or 53BP1 (lower panel). (C & D) Quantitation of the percentage of G1 cells with more than three **C.** RPA or **D.** CtIP foci after the indicated treatments. At least 50 nuclei were counted. (**) indicates *P < 0.001*, and (ns) means *P > 0.05.* Shown are the mean ±SEM for three independent experiments.

DSB end resection is a two-step process. Whereas MRE11 and CtIP are required for the initial limited end resection to generate a short track 3′-ssDNA overhang, EXO1 and DNA2 are required for the progression of end resection to produce a long track 3′-ssDNA, which therefore promotes HR [33]. Here, we sought to investigate the extent of DSB end resection in 53BP1-depleted G1 cells. To that end, 53BP1 was depleted in asynchronous A549 cells before irradiation with 2Gy. We subsequently (at 2h post-IR) compared the RPA foci intensity in G1 and S/G2 cells. As shown in Figure 4A, RPA forms significantly more intense foci in S/G2 compared to G1 phase *(P = 0.003)*, suggesting a short track DSB-end resection in G1 phase. Consistent with this, depletion of EXO1 (Figure 4A, upper panel) did not affect RPA foci intensity in G1 but significantly reduced it in S/G2 (Figure 4A, lower panel). Moreover, knockdown of CtIP or MRE11 (Figure 4B) but not EXO1 diminished the formation of RPA foci in 53BP1-depleted G1 cells, indicating that end resection is only initiated but not extended at these DSBs (Figure 4C). As expected, individual depletion of CtIP, MRE11, or EXO1 showed no effect on RPA foci in G1-phase.

Altogether, this data indicates that when 53BP1-mediated end protection is impaired, DSB-ends are minimally resected by MRE11 and CtIP, which hence might explain why HR is not activated at these DSBs.

### Polo-like-kinase 3 (PLK3) is required for the CtIP-mediated end resection in G1 in 53BP1-depleted cells

CtIP was shown to be activated in S/G2 phase in a CDK1/2-dependent manner [34]. Given that CDK1/2 is active solely in S/G2 but not in G1 phase, we sought to address how CtIP can be activated in 53BP1/RIF1-depleted G1 phase cells. Previously, it was shown that Polo-like kinase-3 (PLK3) phosphorylates CtIP in G1-phase at the same CDK1/2 phosphorylation sites, which hence activates its nuclease function [35]. We therefore investigated whether PLK3 is involved in CtIP phosphorylation in G1 after 53BP1 depletion. To this end, after 53BP1 knockdown, number of RPA foci was monitored in G1-phase at 2h post-2Gy in the presence or absence of PLKi GW843682X, at a concentration of 9.1 nM to specifically inhibit PLK3 [35]. Importantly, inhibition of PLK3 diminished the formation of RPA foci in 53BP1-depleted G1 cells (Figure 4C). Similarly, siRNA-mediated PLK3 knockdown significantly reduced the number of RPA foci in G1 cells after 53BP1 depletion. Consistently, CtIP recruitment was significantly decreased after PLK3 inhibition or knockdown (Figure 4D). Together, these data indicate that PLK3 is required for the CtIP-mediated end resection in G1-phase after depletion of 53BP1.

### PARP1-dependent end joining repairs the resected DSBs in 53BP1- or RIF1-depleted G1 cells

**Figure 5:**
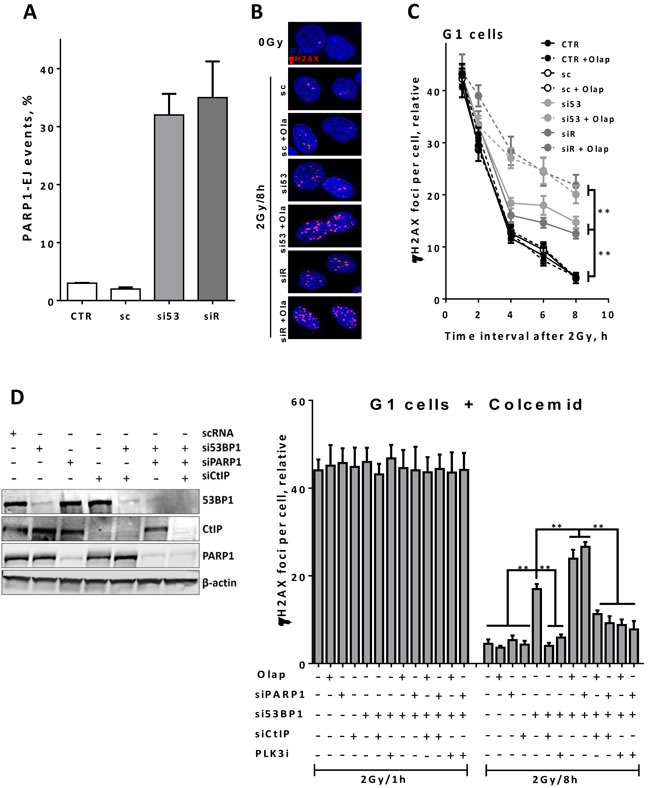
PARP1-dependent end joining repairs the resected DSBs in 53BP1- or RIF1-depleted G1 cells **A.** HeLa cells harboring pEJ were treated with the indicated siRNAs for 24h and PARP1 was then inhibited by adding 1μM olaparib 2h before transfection with I-SceI-expressing vector. The percentages of GFP^+^ cells were measured after 48h as indication for end joining efficiency. PARP1-EJ was measured as the percent of suppressed end joining after PARP1 inhibition. **B.** Representative micrographs for γH2AX foci at 8h post 2Gy in A549 cells after depletion of either 53BP1 or RIF1 proteins in the presence or absence of olaparib. Nuclei were counterstained with CenpF to determine G1 (CenpF^−^) cells. **C.** Quantitation of γH2AX foci number at the indicated time points in CenpF^−^ G1 cells. **D.** Left panel: Western blot showing an efficient siRNA-mediated knockdown of the indicated individual or combined proteins. Right panel: Asynchronous A549 cells were treated with colcemid (0.2μg/ml) for 1h pre-2Gy and the number of γH2AX foci was monitored in CenpF^−^ G1 cells after the indicated treatment conditions. At least 50 nuclei were counted. (**) indicates *P < 0.001*. In all cases, the number of foci measured in non-irradiated cells was subtracted (relative). Shown are the mean ±SEM for three independent experiments. CTR: control, Olap: olaparib, sc: scrambled RNA, si53: si53BP1, and siR: siRIF1.

Previously, we and others have verified an alternative end joining pathway, which depends on PARP1 (PARP1-EJ) and favors working on resected DSB ends [5–7, 36]. Therefore, we first tested whether depletion of 53BP1 or RIF1 causes a switch to PARP1-EJ. To this end, we sought to use the end joining substrate pEJ to measure end joining efficiency in the presence or absence of PARPi olaparib. As illustrated in Figure 5A, PARP1-EJ is not involved in the repair of DSB in non-depleted HeLa cells. However, upon depletion of 53BP1 or RIF1 we observed an increase in the switch to PARP1-EJ. Next, we verified whether the PARP1-EJ pathway operates on resected DSBs in G1-phase in 53BP1- or RIF1- depleted cells. In order to address this posit, we inhibited PARP activity using olaparib in asynchronous A549 cells and monitored the γH2AX foci kinetics in CenpF^−^ 53BP1- or RIF1-depleted cells after 2Gy. In control cells, γH2AX foci numbers in G1 were not significantly affected after olaparib treatment (Figure 5B & 5C), indicating that PARP1-EJ is not involved in the repair of IR-induced DSBs under normal conditions. However, 53BP1- or RIF1- depleted cells showed significantly elevated numbers of γH2AX foci in G1 after PARP inhibition, demonstrating that PARP inhibition precludes the repair events reported in G1 cells after depletion of 53BP1 or RIF1. Similarly, PARP1 depletion (Figure 5D, left panel) showed more residual γH2AX foci (i.e. at 8h) in G1 cells (Figure 5D, right panel). In order to ensure that cells measured at the indicated time points in G1-phase had also been initially irradiated in G1 and not passed over from M-phase, we treated the cells with the mitotic spindle inhibitor colcemid for 1h pre-2Gy to inhibit the exit from M-phase, and subsequently monitored γH2AX foci at 1h and 8h post-2Gy in 53BP1-depleted G1 cells. Confirming the above data, we found that PARP1 inhibition as well as depletion significantly increased *(P < 0.001)* the number of residual γH2AX foci compared to control cells (Figure 5D), indicating a switch to PARP1-EJ. Intriguingly, inhibition of end resection (i.e. by CtIP-knockdown) rescued the repair in 53BP1-depleted G1 cells and prevented the switch to PARP1-EJ as evidenced by a significant decrease *(P < 0.001)* in the number of γH2AX foci at the 8h time point (Figure 5D) after synergistic depletion of 53BP1 and PARP1. In line with this, PLK3 inhibition rescued NHEJ in G1-phase and prevented the switch to PARP1-EJ in 53BP1-depleted cells (Figure 5D). Collectively, these data together with the data presented in Figure 5 demonstrate that PARP1-EJ but not HR (probably due to limited end resection) acts on the resected DSB ends (mediated by CtIP/MRE11) in G1-phase after knockdown of end protection factors 53BP1 or RIF1.

## DISCUSSION

The process of choosing the appropriate DSB repair pathway is of great importance for the maintenance of genomic integrity. For instance, the engagement of NHEJ in replication- associated DSBs is linked to genomic rearrangements and cell death [37]. On the other hand, the activation of HR in G1 phase may lead to loss of heterozygosity and chromosomal translocations [1, 38, 39]. Therefore, the choice of the appropriate DSB repair pathway must be tightly controlled. In the current study, we did not only confirm previously published data [16, 17, 19, 20] but also further extended our understanding of the regulatory role of DDR signaling proteins 53BP1-RIF1 in repair pathway choice. In particular, we verify that RIF1 is a downstream effector for 53BP1 (Figure 1F) and that its loss phenocopies 53BP1 deficiency in terms of enhanced BRCA1 and CtIP recruitment (Figure 1B & S2B), increased RPA and RAD51 loading (Figure 1C & 1D), thus confirming that 53BP1 and RIF1 are effective barriers against DSB end resection in G1 [12, 14, 20, 40]. Consequently, opposing activities were reported between 53BP1-RIF1 on one hand and BRCA1-CtIP on the other hand to regulate DSB repair pathway choice: In G1, 53BP1-RIF1 succeeds in limiting the recruitment of BRCA1 and CtIP, thus committing the repair to NHEJ. However, in S/G2, BRCA1 and CtIP are more efficient, likely because of the activation of CDK1/2 and/or the enhanced displacement of active ATM from the vicinity of DSBs (Figure 2), and remove 53BP1 and RIF1 from DSBs (Figure 2 & [12]) to facilitate end resection, switching the repair to HR (Figure 1A).

Importantly, we demonstrate here that suppression of 53BP1-RIF1 stimulates resection of DSB-ends in G1-phase. Interestingly, this end resection was shown to depend on CtIP/MRE11 but not EXO1, which indicates a short-track DSB end resection in G1 in the absence of 53BP1/RIF1 protection. In accord with this, we report that RPA foci intensity, as a measure for the extent of end resection, is lower in G1 compared to that in S/G2 cells (Figure 4A). Noteworthy, a recent study identified HELB as a feedback inhibitor for the EXO1-, DNA2- and BLM-mediated end resection in G1-phase [41], which may explain why we observed only short-track end resection in G1 cells in the absence of 53BP1/RIF1, which was not sufficient to stimulate HR. Consistently, we report that 53BP1 or RIF1 depletion enhances the recruitment of BRCA1, CtIP and RPA (Figure 3A-3C) but importantly fails to cause RAD51 recruitment (Figure 3D) in G1-phase. Notably, a recent study from the Durocher lab has reported that RAD51 loading to DSB is prevented in G1 by antagonizing the deubiquitylase USP11 and hence preventing BRCA1-PALB2-BRCA2 binding [42].

It has been shown that CtIP is phosphorylated and activated by PLK3 in G1, which may lead to end resection [35]. In line with this, we show here that end resection in G1 is indeed mediated by PLK3, as CtIP and RPA foci were diminished after inhibition or depletion of PLK3 (Figure 4C & 4D). Intriguingly, we showed here for the first time that after 53BP1 or RIF1 depletion, the resected DSBs in G1 are processed by PARP1-EJ and that the switch to PARP1-EJ is suppressed upon inhibition of PLK3-CtIP-mediated end resection (Figure 4 & 5). Noteworthy, CtIP-mediated end resection in G1-phase was previously reported for complex DSBs in Ku-knockout cells after α-particle irradiation [35].

Based on the data presented in the current work, the activity of RIF1 in blocking end resection can be explained by two mechanisms. The first mechanism is due to the existence of the DNA binding domain in the C-terminal of RIF1, which could account for RIF1-mediated end protection activity. Interestingly, *in vitro* studies indicate that this domain strongly binds to double stranded DNA (dsDNA) but not to single stranded DNA (ssDNA) [43]. Upon induction of DSBs after IR, the presence of dsDNA around DSB provides a docking station for the recruitment of RIF1, which prevents the access of the ends to end processing proteins BRCA1 and CtIP. This explains why depletion of RIF1 renders DSB ends accessible by BRCA1, CtIP, RPA, and RAD51 proteins (Figure 1B-1D and S2B). When DSB ends are resected and ssDNA stretches become longer, RIF1 cannot prevent the recruitment of CtIP, RPA, RAD51 to DSB ends due to its weak ssDNA binding activity. In line with this assumption, immunoprecipitation data show that yeast rif1 binds at short but not long ssDNA which in turns blocks the recruitment of RPA [44, 45]. This explains why RIF1 forms stronger (more intense) foci in G1 where end resection is limited and weaker foci in S/G2 where end resection is activated (Figure S2A). The second mechanism relies on the fact that 53BP1-mediated end protection activity requires its ability to oligomerize and bind to the abundant H4K20me2 [32]. Perhaps cooperative interaction between 53BP1 and RIF1 acts to stabilize 53BP1 oligomers at DSBs, keeping the chromatin state around DSB refractory to access by nucleases. In support of this assumption, we showed that 53BP1 IRIF are diminished in intensity when RIF1 is depleted [12]. Thus, the establishment of a 53BP1-RIF1 mediated chromatin barrier in the vicinity of DSBs preserves their integrity, favoring NHEJ and preventing error-prone repair pathways. Noteworthy, PTIP is also a downstream effector of 53BP1 and was reported to be involved in protection of DSB ends from resection. PTIP and RIF1 are independently recruited to DSBs in a phospho-53BP1-dependent manner, as they exhibit distinct phosphorylation-dependent interactions with 53BP1 that guide them to DSBs [15]. However, the exact mechanism for the crosstalk between the two proteins or whether they bind simultaneously to 53BP1 is still unclear. A suggested model is a collaborative interactions between RIF1 and PTIP in regulating the end resection, for example, RIF1 appears to be involved in protection against initial but not sustained resection ([19] & our unpublished data), however whether PTIP regulates the sustained end resection is still to be elusive.

**Figure 6:**
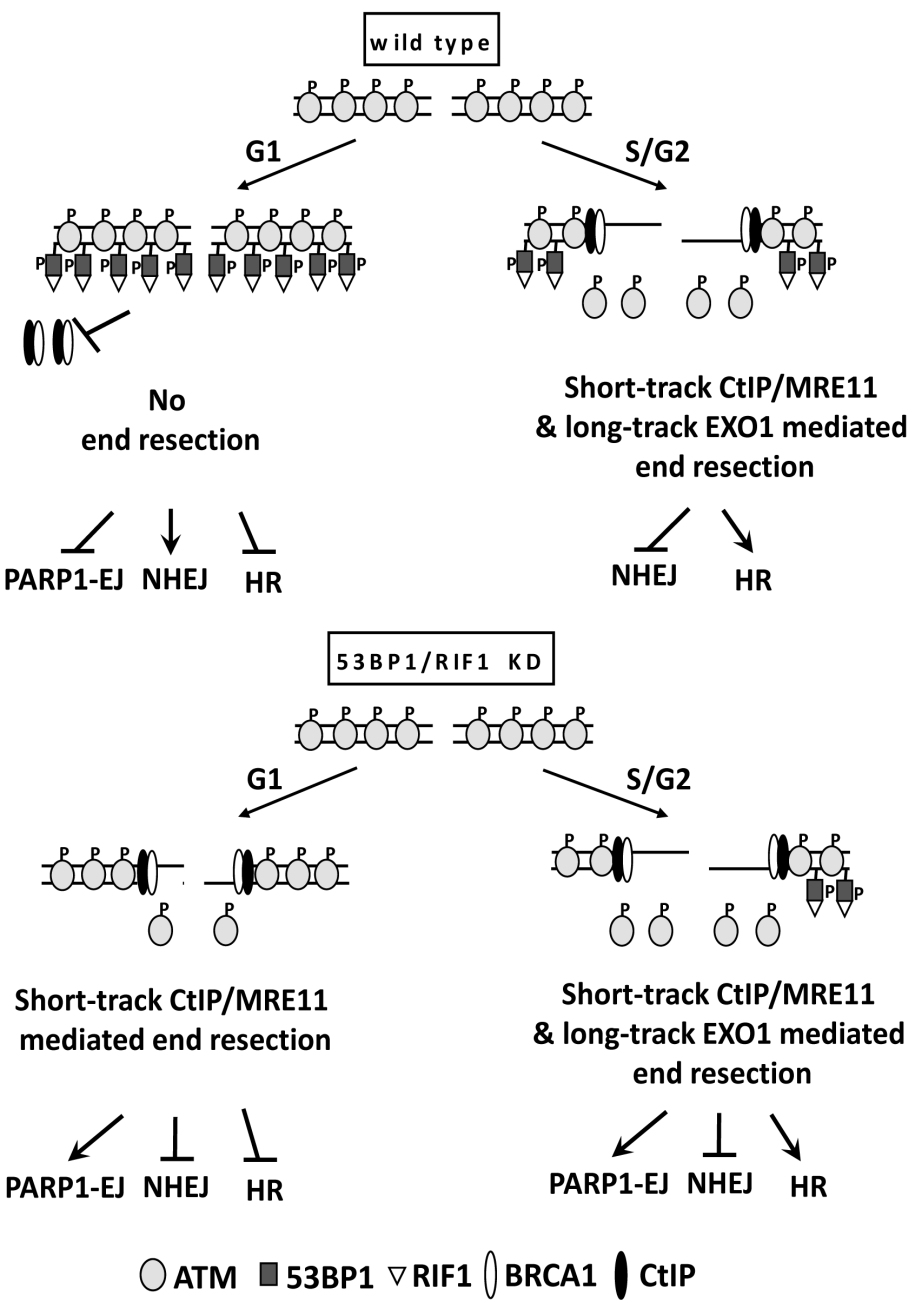
A proposed model for regulation of DSB repair pathway choice by ATM-mediated 53BP1-RIF1 end protection In wild type cells, activated ATM (i.e. after DSB induction) phosphorylates 53BP1 to stimulate RIF1 recruitment. 53BP1/RIF1 suppresses BRCA1/CtIP- mediated end resection and promotes NHEJ. Activation of end resection in S/G2 phase displaces pATM, decreases 53BP1 phosphorylation, which further alleviates the barrier to DSB resection (i.e. short and long track end resection) to commit the repair to HR. In the absence of 53BP1/RIF1, end resection is further enhanced in S/G2-phase, leading to stimulation of HR and PARP1-EJ. In 53BP1-depleted G1 cells, end resection is activated properly, due to PLK3 mediated CtIP phosphorylation. However, EXO1 cannot process these DSBs, leading therefore to only short-track end resection, which is not sufficient to stimulate HR but instead switches the repair to the alternative PARP1-EJ.

In conclusion, as shown in Figure 6, we report here that in wild type cells, upon DSB induction and activation of ATM, chromatin-bound activated ATM phosphorylates 53BP1, which stimulates the recruitment of and the binding to RIF1. 53BP1/RIF1 promotes NHEJ by suppressing the recruitment of BRCA1/CtIP and hence preventing end resection. As cells enter S/G2 phase, end resection is activated *via* CDK1/2 mediated CtIP phosphorylation, which displaces pATM from DSBs, which concurs with a decrease in the phosphorylation of 53BP1 at DSB sites. This consequently attenuates the binding between 53BP1 and RIF1 and further alleviates the barrier to DSB resection (i.e. short and long track end resection) to commit the repair to HR. In accord with this, ATM binding affinity to breaks has been reported to be attenuated with progressive presence of ssDNA at resected DSB [46]. Since only a specific fraction of DSBs is repaired by HR in S/G2 phase, we think that ATM displacement and hence DSB repair choice needs an additional regulatory signal, which might be related to chromatin modulation. Recently, ATM was shown to phosphorylate the histone modifier MOF, which then modulates ATM retention at DSB sites. Interestingly, MOF is hyper-phosphorylated in S/G2, which coincides with the loss of 53BP1 from the DSB ends [47]. This might control 53BP1 function to regulate the subsequent recruitment of HR repair proteins during S/G2 phase.

On the other hand, in the absence of 53BP1/RIF1, the situation in S/G2 phase is not changed; only more DSBs are repaired by HR and PARP1-EJ, due to enhanced DSB end resection. However, in 53BP1- or RIF1-depleted G1 cells, end resection is activated properly through PLK3 mediated CtIP phosphorylation. However, EXO1 cannot process these DSBs, probably due to the nuclear localization of its negative regulator HELB [41], leading therefore to a short-track end resection, which inhibits NHEJ but is not sufficient to stimulate HR and instead switches the repair to the alternative PARP1-EJ.

Finally, the results of the current study are of clinical importance in relation to BRCA1-deficient tumors. The most promising strategy for treating BRCA-deficient malignancies is the use of PARP inhibitors (PARPi) [48]. Although a significant fraction of those patients respond to PARPi, some patients become resistant to this treatment [49, 50]. The fact that 53BP1 or RIF1 depletion enables BRCA1-deficient cells to tolerate PARP inhibition highlights the importance of 53BP1 and RIF1 status as molecular biomarkers for the response to PARPi in BRCA1-mutated malignancies. Breast cancer patients with BRCA1 mutations frequently showed low levels of 53BP1 expression [51]. Interestingly, some PARPi-resistant tumor patients showed restored HR activity without any loss of 53BP1 [51]. Based on the data presented here, we envision that 53BP1 hypo-phosphorylation and the associated failure of RIF1 recruitment may emerge as novel indicators for PARPi resistance in BRCA1-deficient cancers. Moreover, missense mutations in RIF1 have been found in numerous malignancies (http://cancer.sanger.ac.uk/cosmic/gene/analysis?ln = RIF1), and RIF1 protein levels are significantly higher in cancerous lesions than those in benign lesions [52]. This observation highlights the possibility of using RIF1 as a marker for predicting the response to olaparib.

## MATERIALS AND METHODS

### Cell culture, X-irradiation and inhibitors

The human cervical carcinoma cell lines HeLa, HeLa-pEJ (harboring the end-joining substrate) HeLa-pGC (containing the gene conversion substrate) and the human lung carcinoma cell line A549 were cultured in Dulbecco's modified Eagle medium (DMEM; Gibco-Invitrogen) supplemented with 10% FCS. The human breast cancer cell line HCC1937 was cultured in RPMI medium (RPMI; Sigma Aldrich) supplemented with 20% FCS. Irradiation was performed as previously described (200 kV, 15 mA, additional 0.5mm Cu filter at a dose rate of 0.8 Gy/min) [53]. 10μM KU55933 and 5μM NU2670 were used to inhibit the kinase activity of ATM and DNAPK, respectively. To inhibit PARP activity, we used 1μM olaparib. PLK3 was inhibited using 9.1nM GW843682X (Sigma-Aldrich).

### RNA interference

siRNAs employed in this study were SMARTpools (ThermoFisher) except for the PARP1 siRNA (5′-GGGCAAGCACAGUGUCAA-3′). RNAi transfections were performed using Lipofectamine RNAiMAX (Invitrogen) according to the manufacturer's protocol. Target sequences of the employed SMART-pool siRNAs are listed in Table S1.

### Colony formation assay

For colony formation, cells were seeded and allowed to adhere for 4h before drug treatment and/or irradiation. After 24h, cells were subsequently incubated in drug-free medium for colony formation for 2-3 weeks and thereafter stained with crystal violet. Colonies of 50 cells or more were counted manually and survival curves were derived from triplicates of at least three independent experiments.

### DSB repair reporter assay

To induce DSBs, HeLa cells harboring stably integrated reporter construct for HR (pGC) or for NHEJ (pEJ) were transfected with the I-SceI expression vector pCMV3xnls-I-SceI (1μg) using Fugene HD (Promega) as a transfection reagent. 48 hours after transfection, the cells were assessed for green fluorescence by flow cytometry (FACScan, BD Bioscience).

### Immunofluorescence

Immunofluorescence analyses were performed as previously described [22]. Briefly, cells grown on cover slips were washed once with cold PBS and fixed with 4% para-formaldehyde/PBS for 10 min. Fixed cells were permeabilized with 0.2% Triton X-100/PBS on ice for 5 min. The cells were incubated overnight with primary antibodies: phospho-S139-H2AX (Millipore,23464), RAD51 (Calbiochem PC130), RPA (Santa Cruz Biotechnology, sc-53496), pATM (Rockland, 200301400), RIF1 (Bethyl, A300-569A), 53BP1 (Novus, NB100-305), BRCA1 (Santa Cruz, sc-6954), CtIP (Active Motif, 61141), Anti-CenpF (Lifespan Biosciences, LS-B276 & LS-B3046), p53BP1(S25/29), and p53BP1 (S1778) (cell signaling, 2675 & 2674S). After being washed three times with cold PBS, the cells were incubated for 1 h with secondary anti-mouse Alexa-fluor594 (Invitrogen) at a dilution of 1:500 or anti-rabbit Alexa-fluor488 (Invitrogen) at a dilution of 1:600. The nuclei were counterstained with 4′-6-diamidino-2-phenylindole (DAPI, 10ng/ml). Slides were mounted in Vectashield mounting medium (Vector Laboratories). Immunofluorescence was observed with the Zeiss AxioObserver.Z1 microscope (objectives: ECPlnN 40x/0.75 DICII, resolution 0.44 μm; Pln Apo 63x/1.4Oil DICII, resolution 0.24 μm; EC PlnN 100x/1.3 Oil DICII, resolution 0.26 μm and filters: Zeiss 43, Zeiss 38, Zeiss 49). Semi-confocal images were obtained using the Zeiss Apotome, Zeiss AxioCamMRm and Zeiss AxioVision Software. Analysis of foci intensities was performed using ImageJ software

### Graphs and statistics

Unless stated otherwise, experiments were independently repeated at least three times. Data points represent the mean ±SEM of all individual experiments. Statistical analysis, data fitting and graphics were performed with the GraphPad Prism 6.0 program (GraphPad Software).

## SUPPLEMENTARY MATERIAL TABLE AND FIGURES


