# Supplementary material for: Impaired 53BP1/RIF1 DSB mediated end-protection stimulates CtIP-dependent end resection and switches the repair to PARP1-dependent end joining in G1
Source: Oncotarget. 2016 Aug 2;7(36):57679–93. doi: 10.18632/oncotarget.11023 (PMC5295381; doi:10.18632/oncotarget.11023)
Supplement: Supplementary file 1 [file oncotarget-07-57679-s001.pdf]

Impaired 53BP1/RIF1 DSB mediated end-protection stimulates CtIP-dependent end resection and switches the repair to PARP1-dependent end joining in G1

Supplementary Material

Table S1: SMART-Pool siRNA target sequence. Each SMART-Pool siRNA composes of 4 individual siRNAs that specifically target the indicated sequence in the transcript of interest.

siRNA Target	Target sequence
53BP1	GAGCUGGGAAGUAUAAAUU - GGACUCCAGUGUUGUCAUU<br>GAAGGACGGAGUACUAAUA - GCUAUAUCCUUGAAGAUUU
RIF1	CCUCAAAUGAAAUGCGAAA - UCACGUAGCCCUAAAUUUA<br>GAAUCAAAUCUAAGGACUA - GCAAGUUCCUGAUGAUUUA
BRCA1	CAACAUGCCCACAGAUCAA - CCAAAGCGAGCAAGAGAAU<br>UGAUAAAGCUCCAGCAGGA – GAAGGAGCUUUCAUCAUUC
EXO1	GCACGUAAUCAAGUGAUG - GUAAAUGGACCUACUAACA<br>CCACCUAGGACGAGAAAUA - CGGAAGAGAAGUUUCGUUA
PLK3	GCAUCAAGCAGGUUCACUA - GCGAGAAGAUCCUAAAUGA<br>CAGAAGUGCUGCUGAGACA - GAAAUUGUAUAGCCUUCAU
CtIP	GGAGCUACCUCUAGUAUCA - GAGGUUAUAUUAAGGAAGA<br>GAACAGAAUAGGACUGAGU - GCACGUUGCCCAAAGAUUC
MRE11	GAUGAGAACUCUUGGUUUA - GAAAGGCUCUAUCGAAUGU<br>GCUAAUGACUCUGAUGAUA - GAGUAUAGAUUUAGCAGAA
scRNA	UAGCGACUAAACACAUCAA - UAAGGCUAUGAAGAGAUAC<br>AUGUAUUGGCCUGUAUUAG - AUGAACGUGAAUUGCUCAA

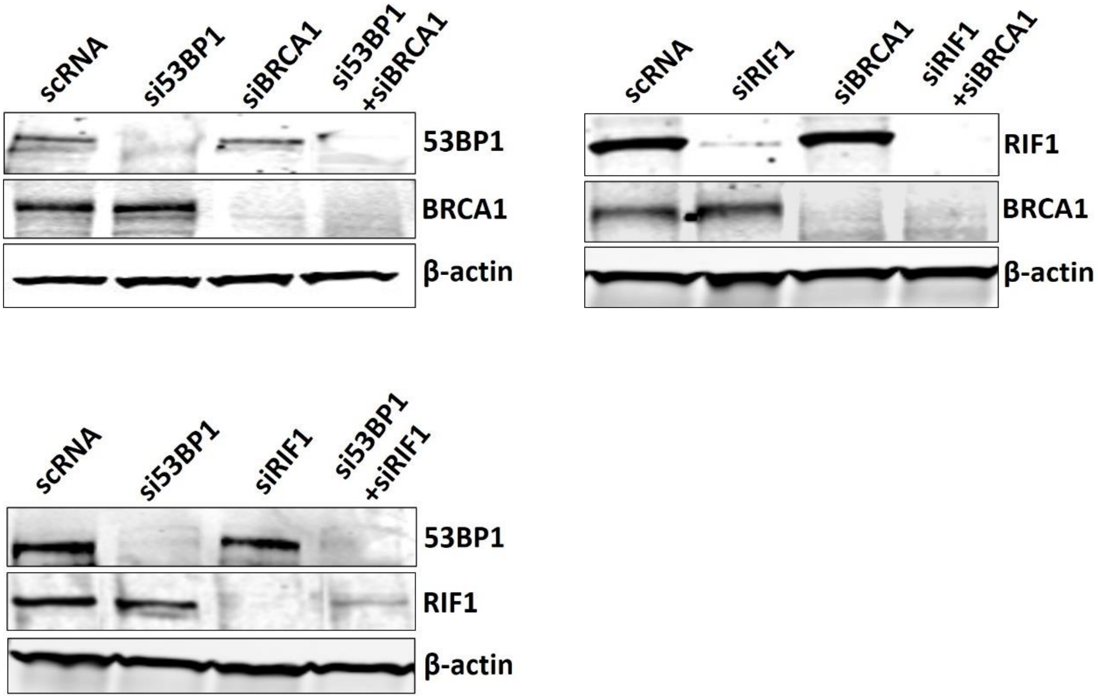

Figure S1. Western blot showing an efficient individual or combined siRNA-mediated knockdown for BRCA1, 53BP1, and RIF1. Cells were transfected with the indicated siRNAs either individually or combined for 48h before collection and protein extraction. About 30μg total protein were electrophoresed and transblotted using Western blot to detect the expression of the indicated protein. β-actin is used as a loading control.

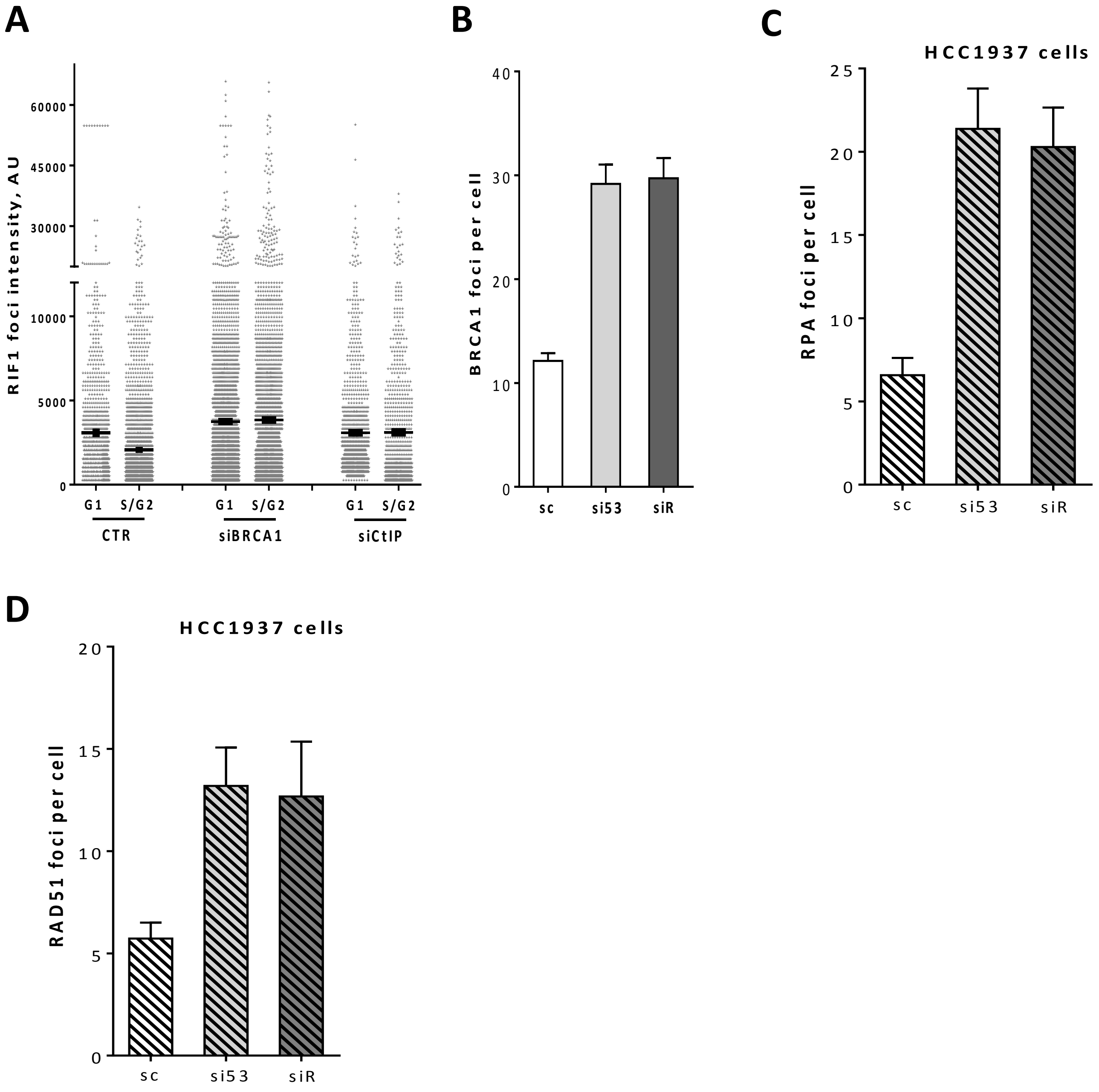

Figure S2. Depletion of either 53BP1 or RIF1 rescues HR in BRCA1-deficient cells. (A) Asynchronous A549 cells were treated with the indicated siRNAs and RIF1 foci intensities were quantified in G1 (CenpF$^-$) and S/G2 (CenpF$^+$) cells at 2h after 2Gy. (B) BRCA1 foci were counted in A549 S/G2 cells treated as in A. The data of scRNA control and si53BP1 depletion were taken from Bakr et al., 2016). (C-D) BRCA1-deficient HCC1937 cells were treated with the indicated siRNAs 48h before irradiation with 2Gy. Cells were then immunostained for RPA (C) and RAD51 (D) after 2h and 4h, respectively. (E) Clonogenic survival fractions of A549 cells treated with the indicated siRNAs before exposure to the indicated doses of olaparib (left panel) or MMC (right panel). Shown are the mean ±SEM for three independent experiments. sc: scrambled RNA, si53: si53BP1, siR: siRIF1, and siB: siBRCA1.

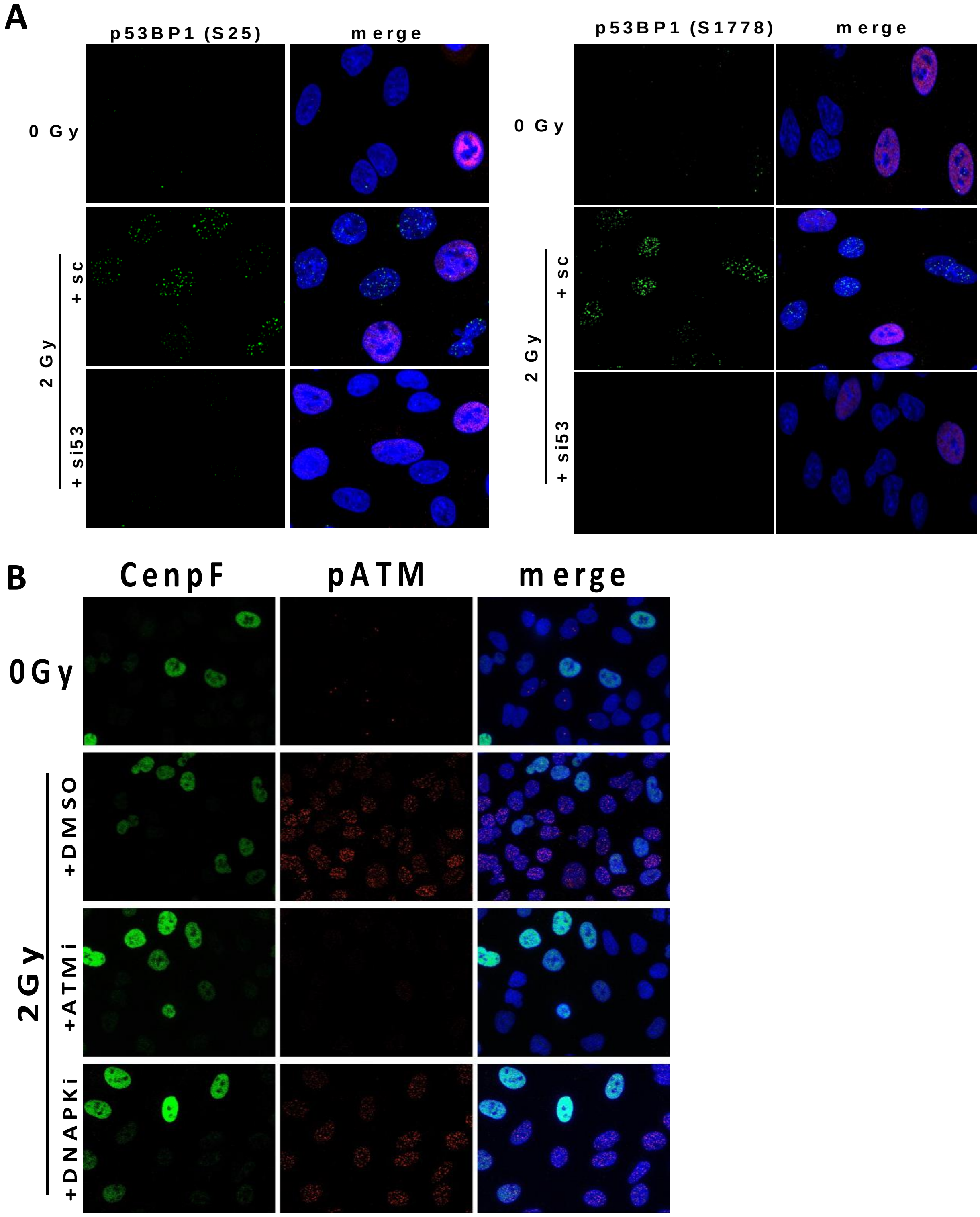

Figure S3. Validation of p53BP1 and pATM antibodies used in this work. (A-B) Asynchronous A549 cells were treated with 10µM KU55933 or 10µM NU7026 to inhibit ATM or DNAPK, respectively for 1h before irradiation with 2Gy and subsequently stained at 1h post-IR for p53BP1 (S25/29) and (S1778) (A) or at 2h post-IR for pATM (B) after the indicated treatments. Anti-CenpF staining was used to distinguish between G1 and G2 phases. sc: scrambled RNA, si53: si53BP1. ATMi: ATM inhibitor KU55933. DNAPKi: DNAPK inhibitor NU7026.

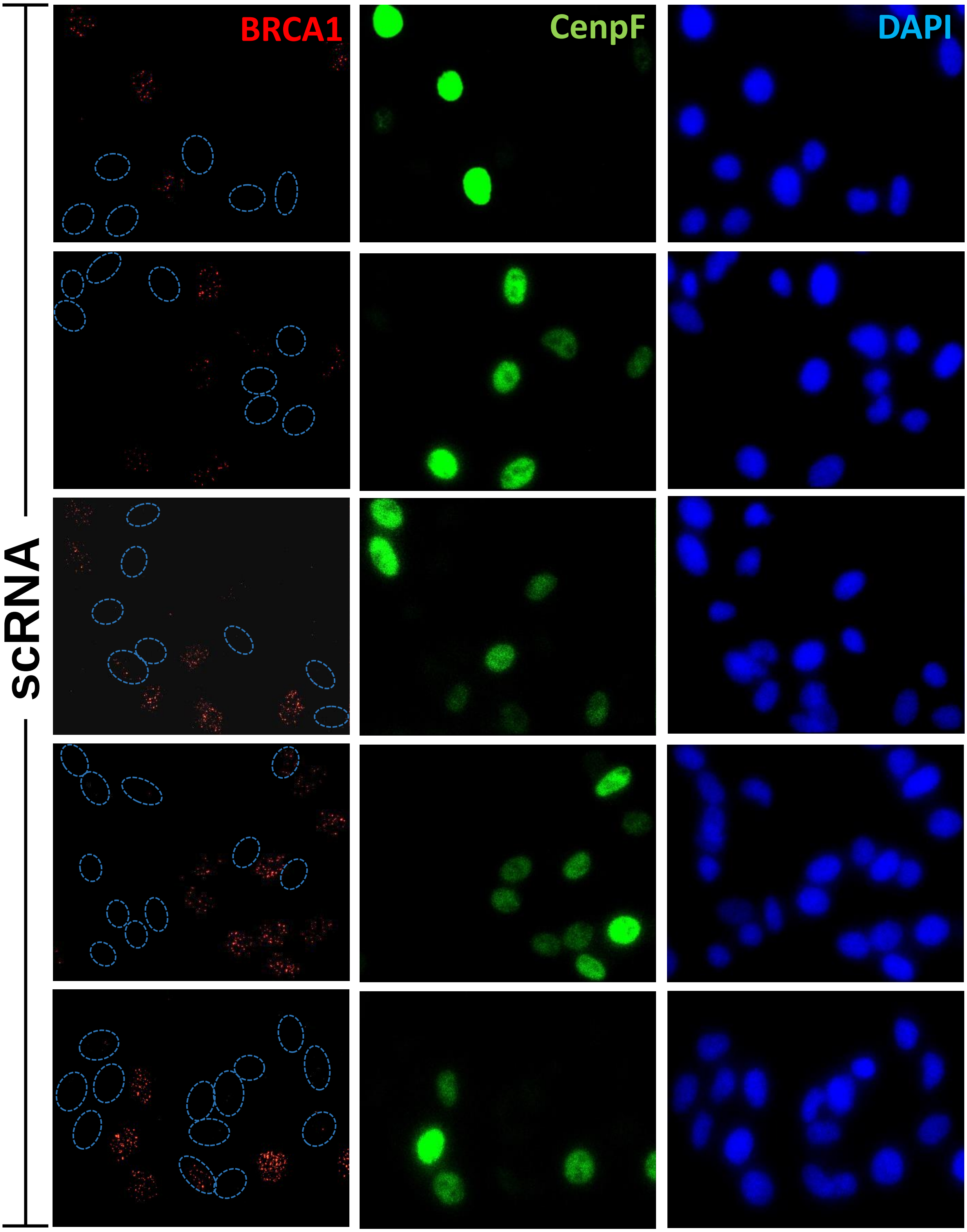

Figure S4A

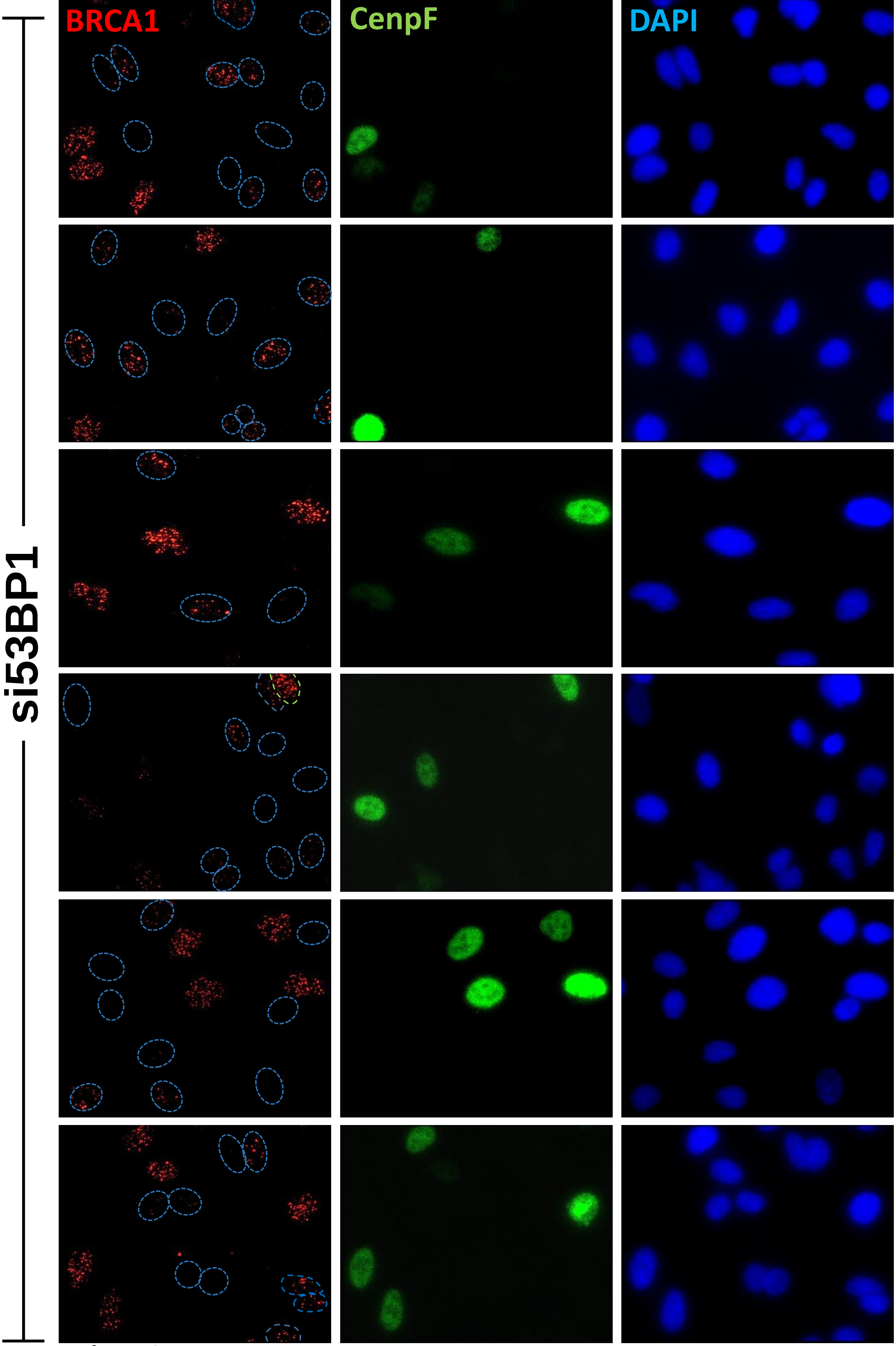

Figure S4B .

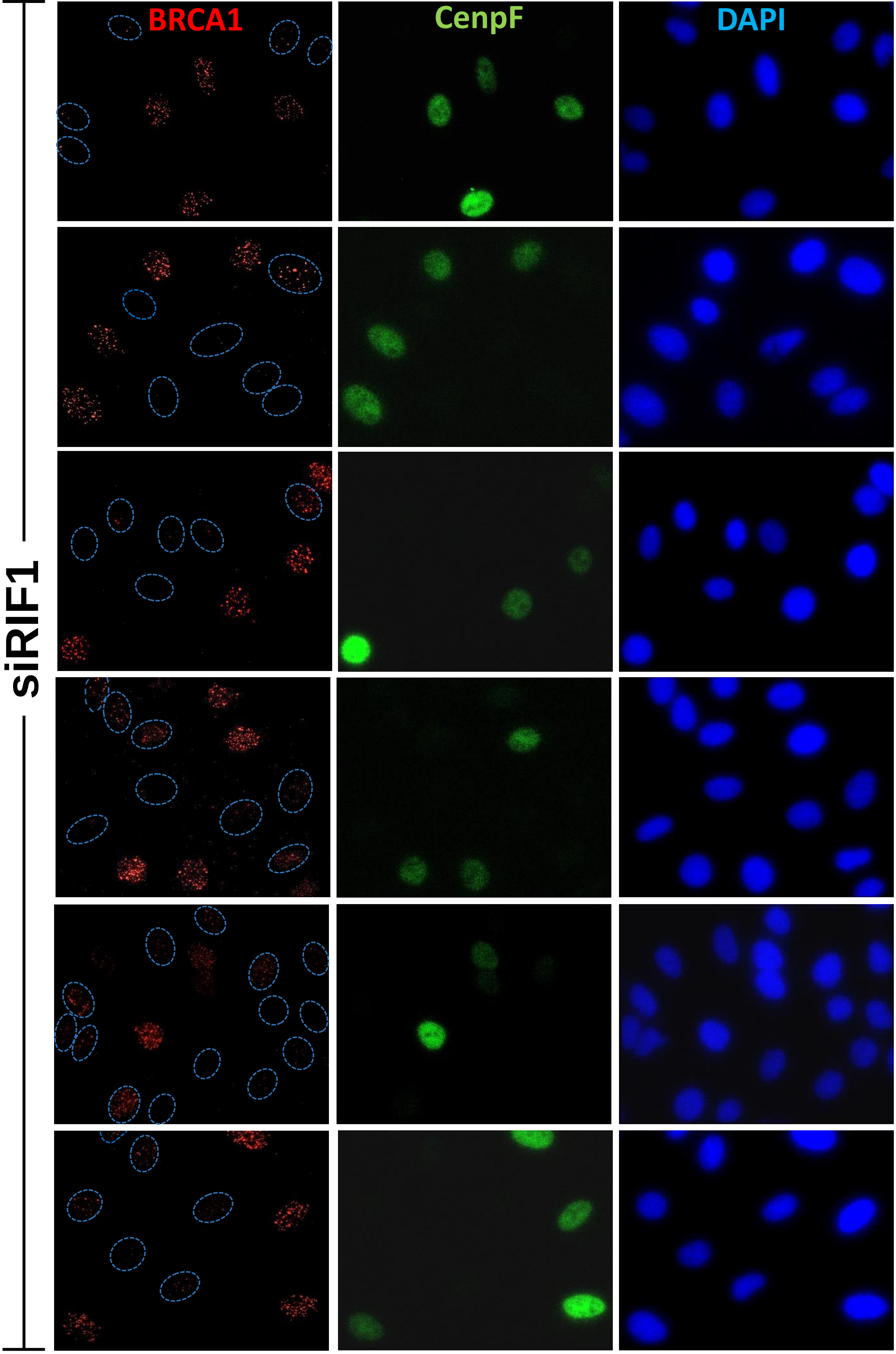

Figure S4C.

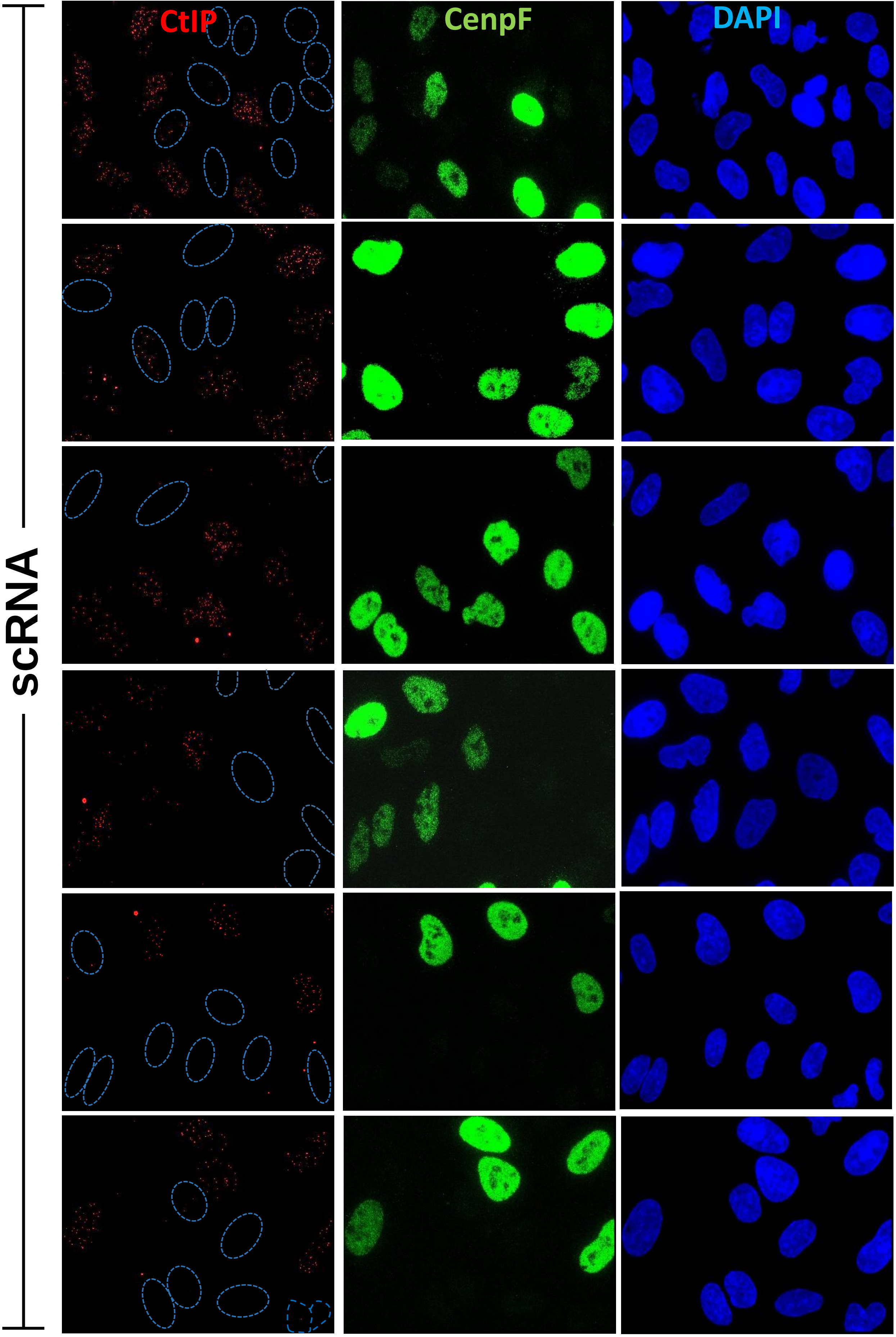

Figure S4D.

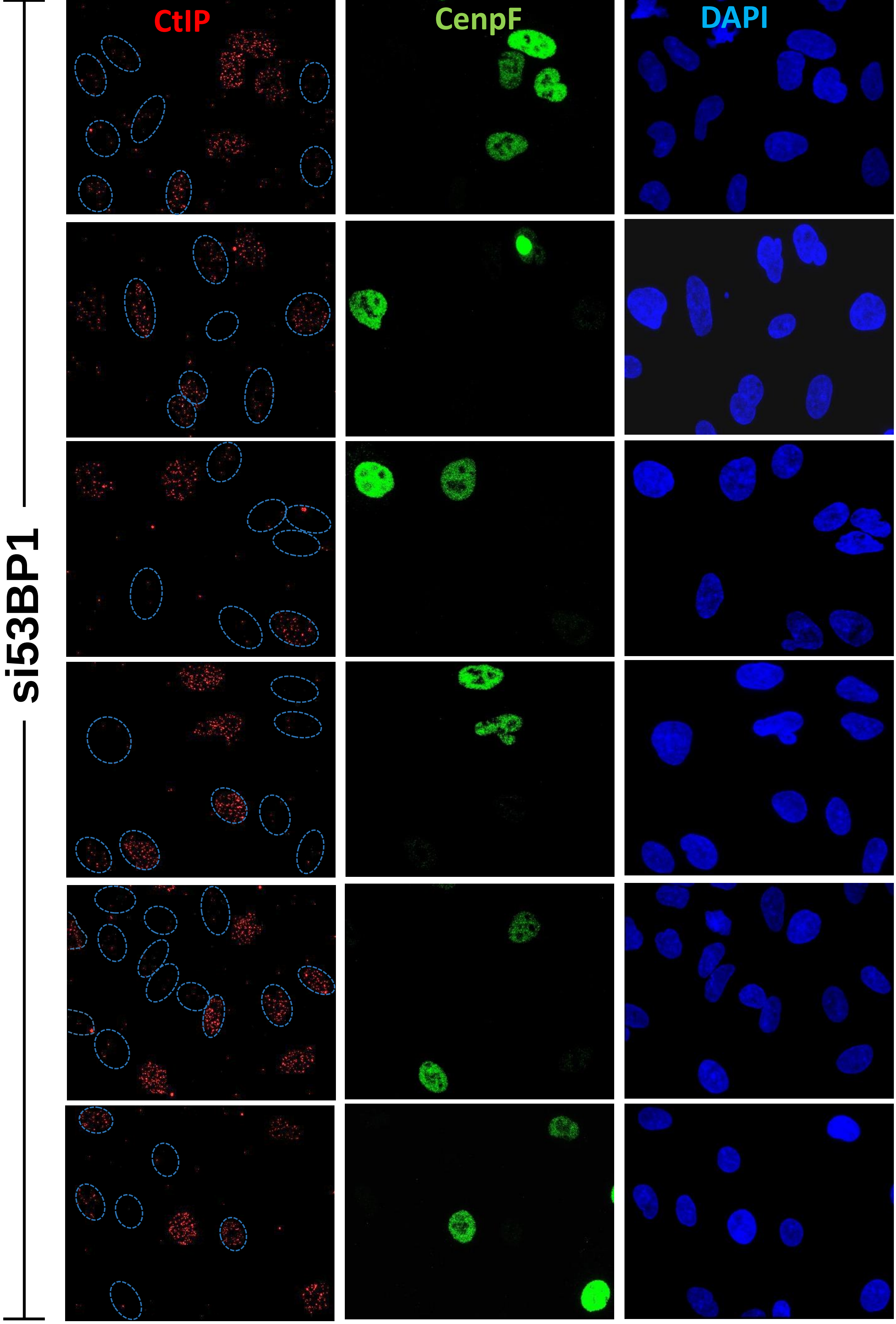

Figure S4E.

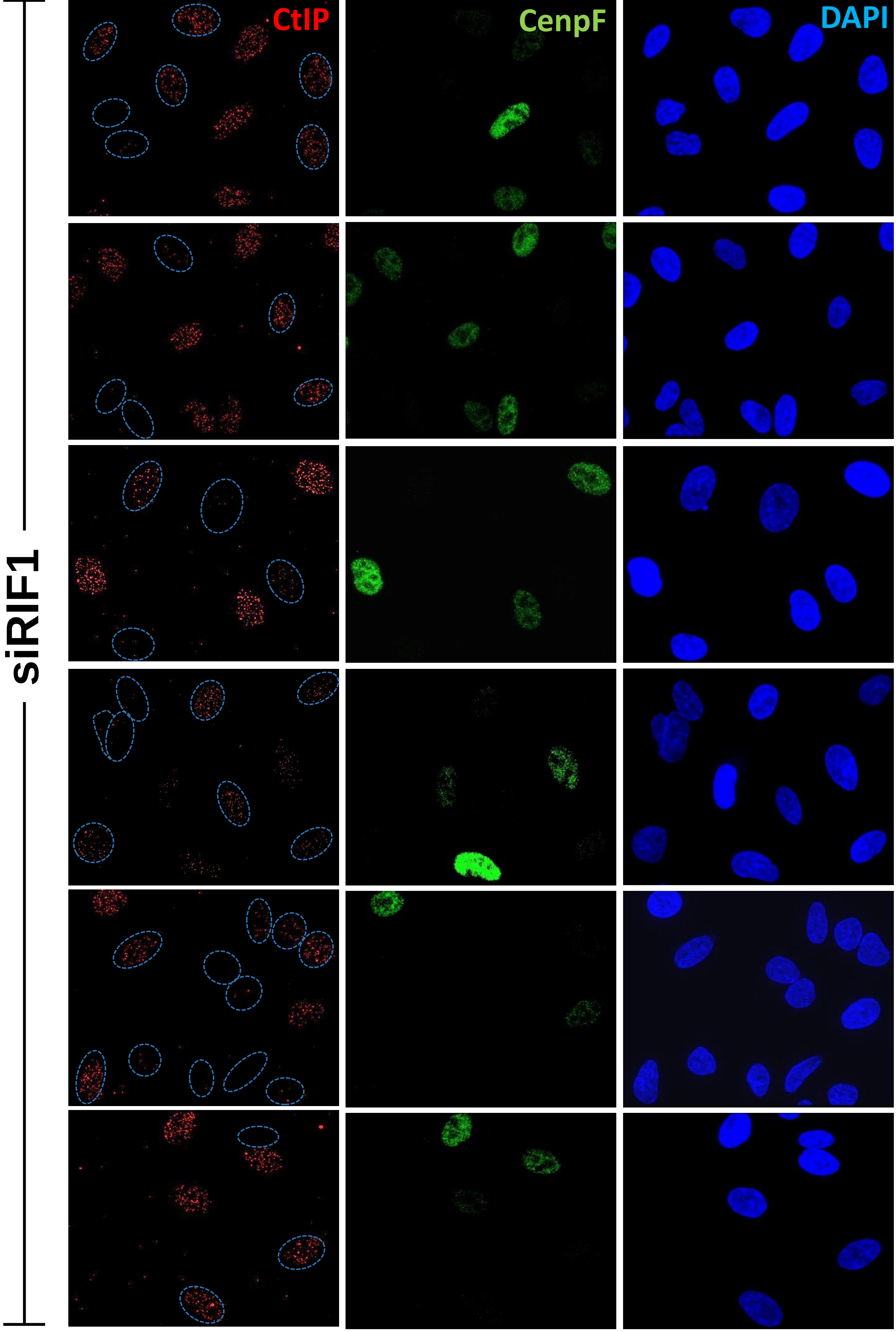

Figure S4F

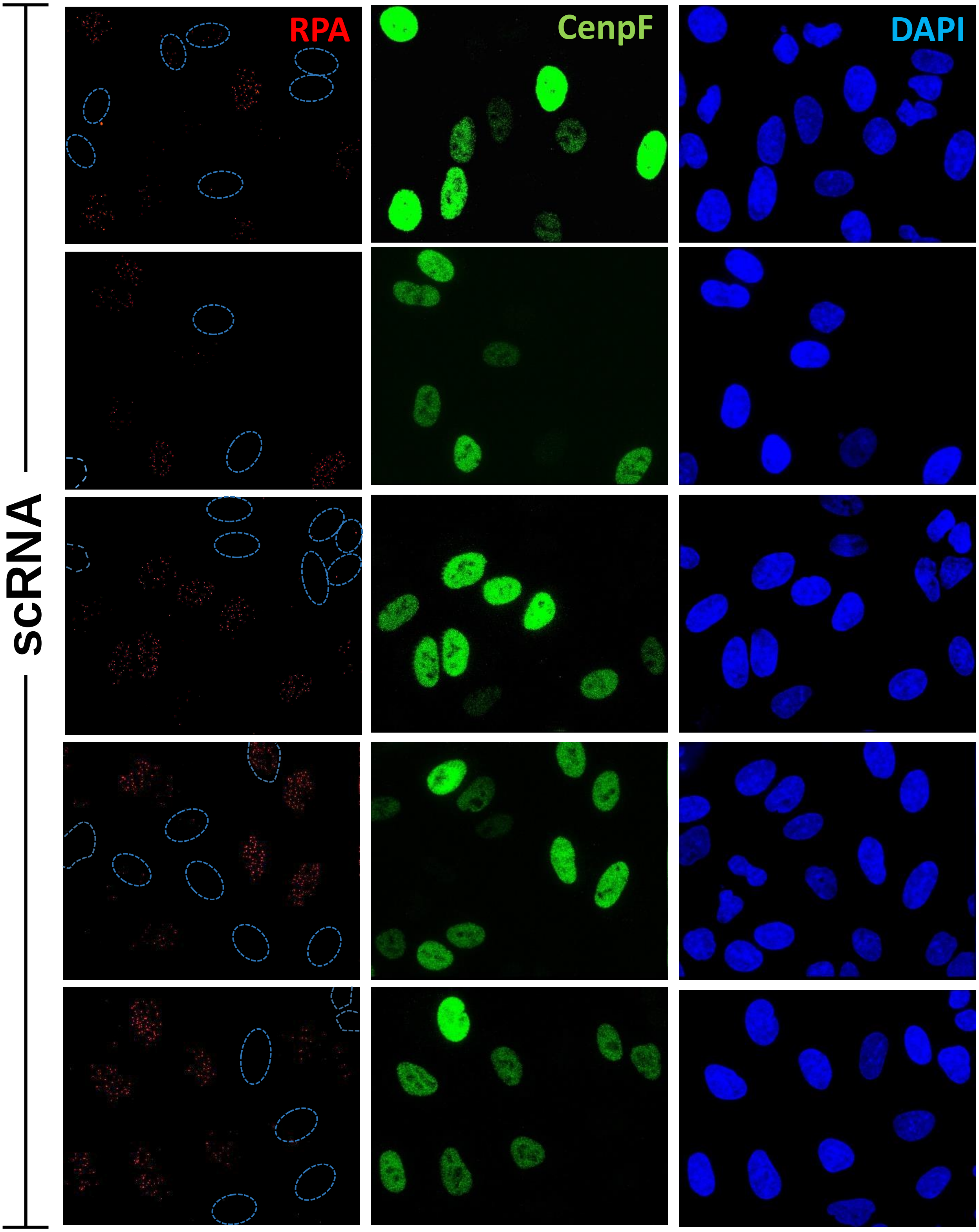

Figure S4G.

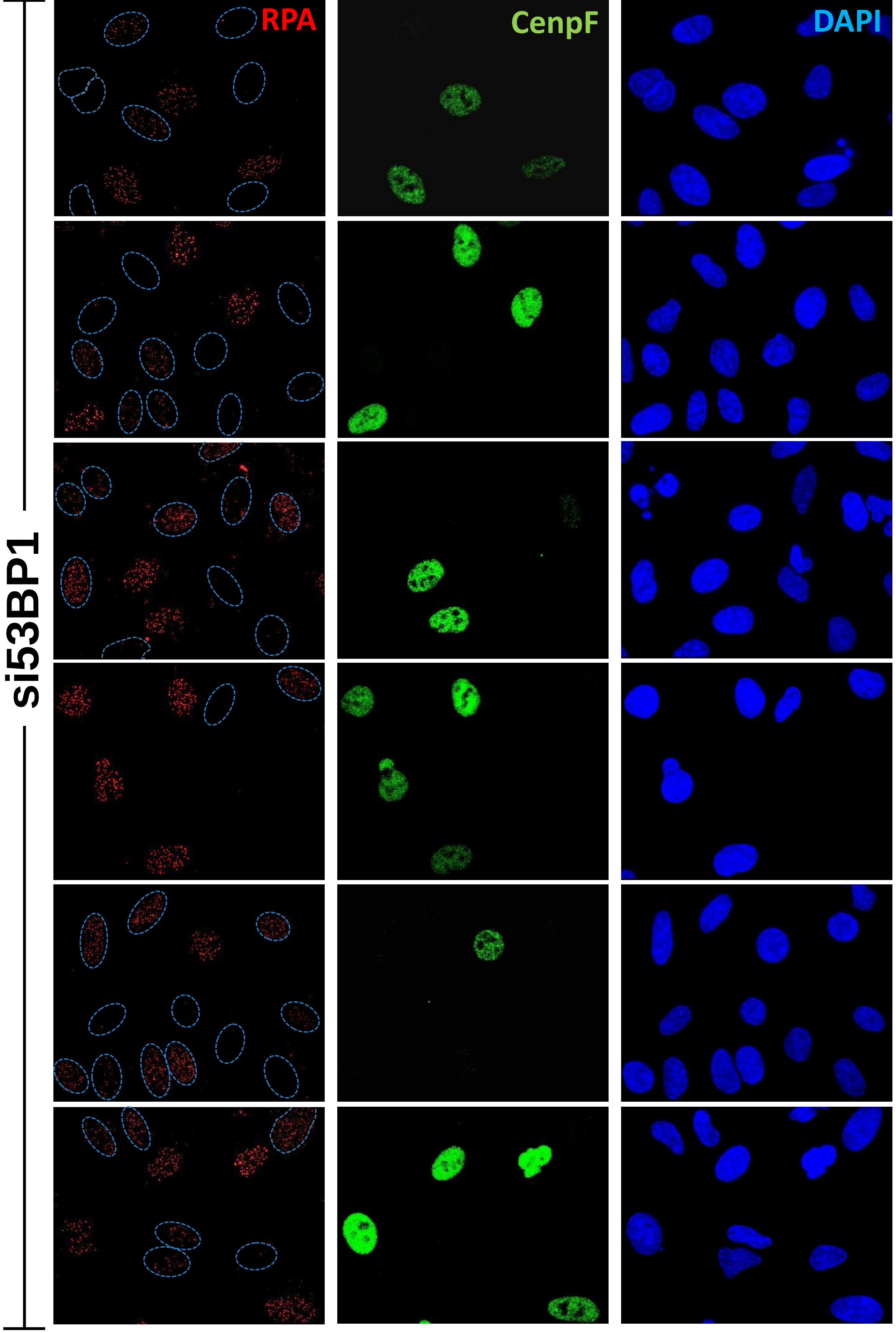

Figure S4H

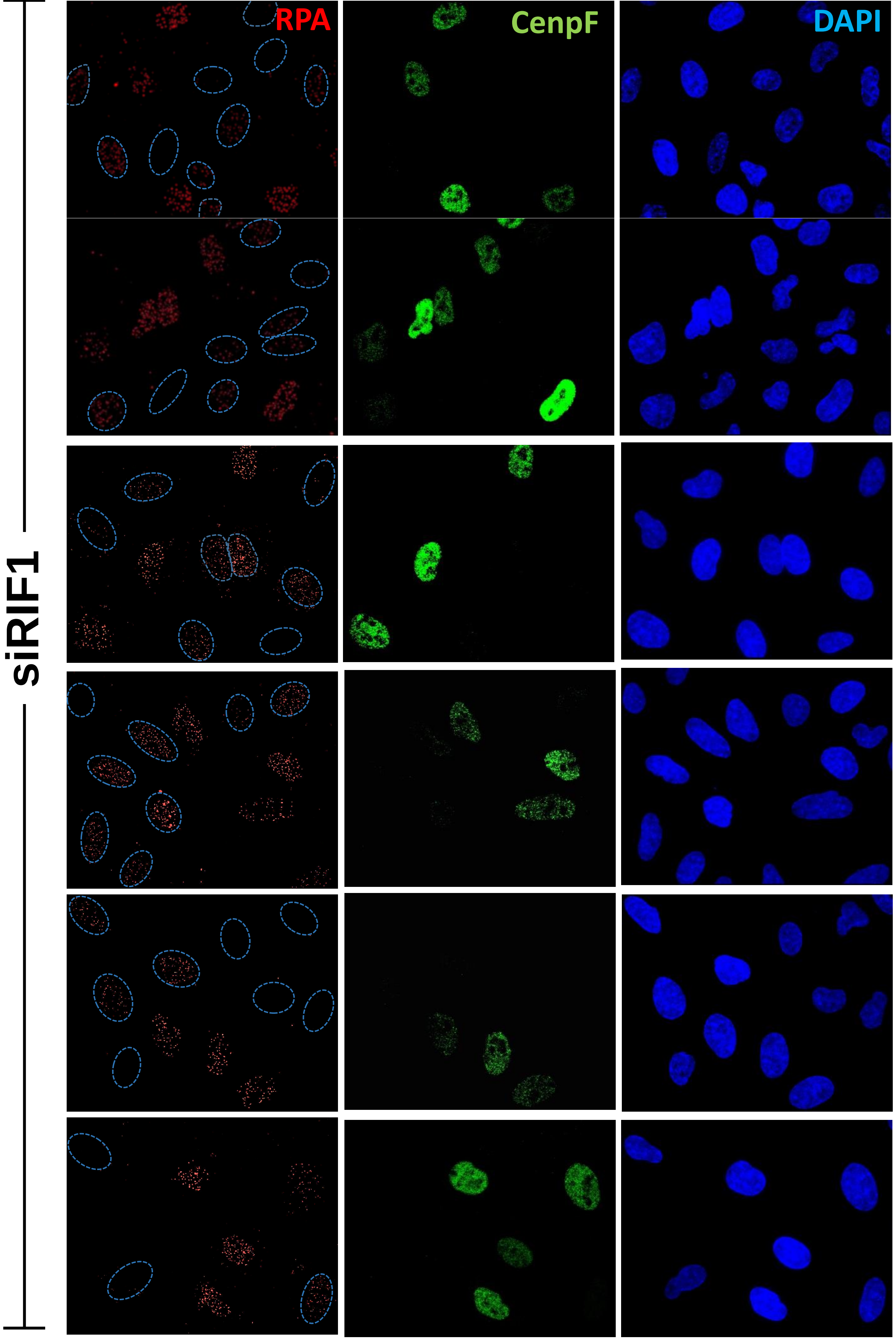

Figure S4I

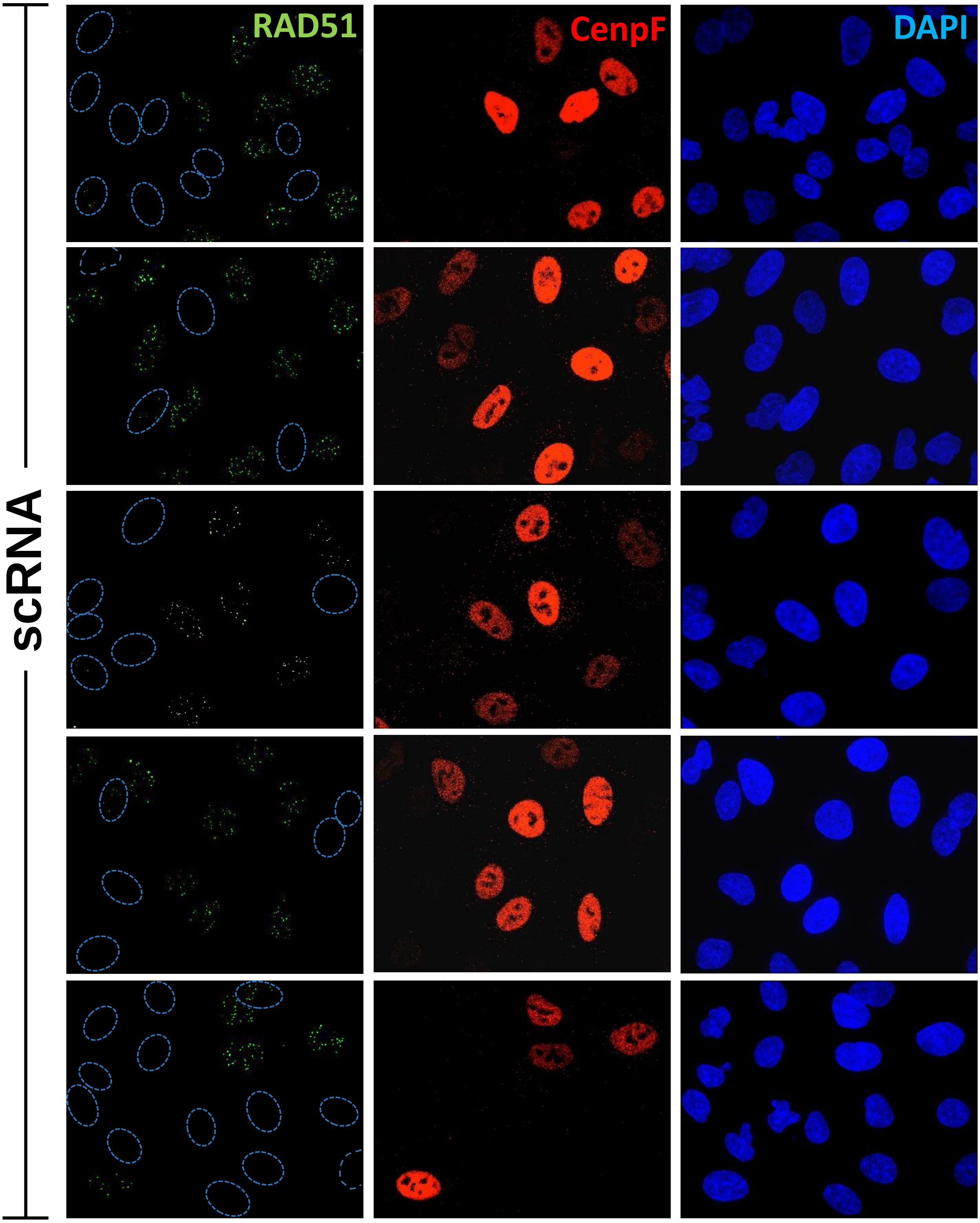

Figure S4J.

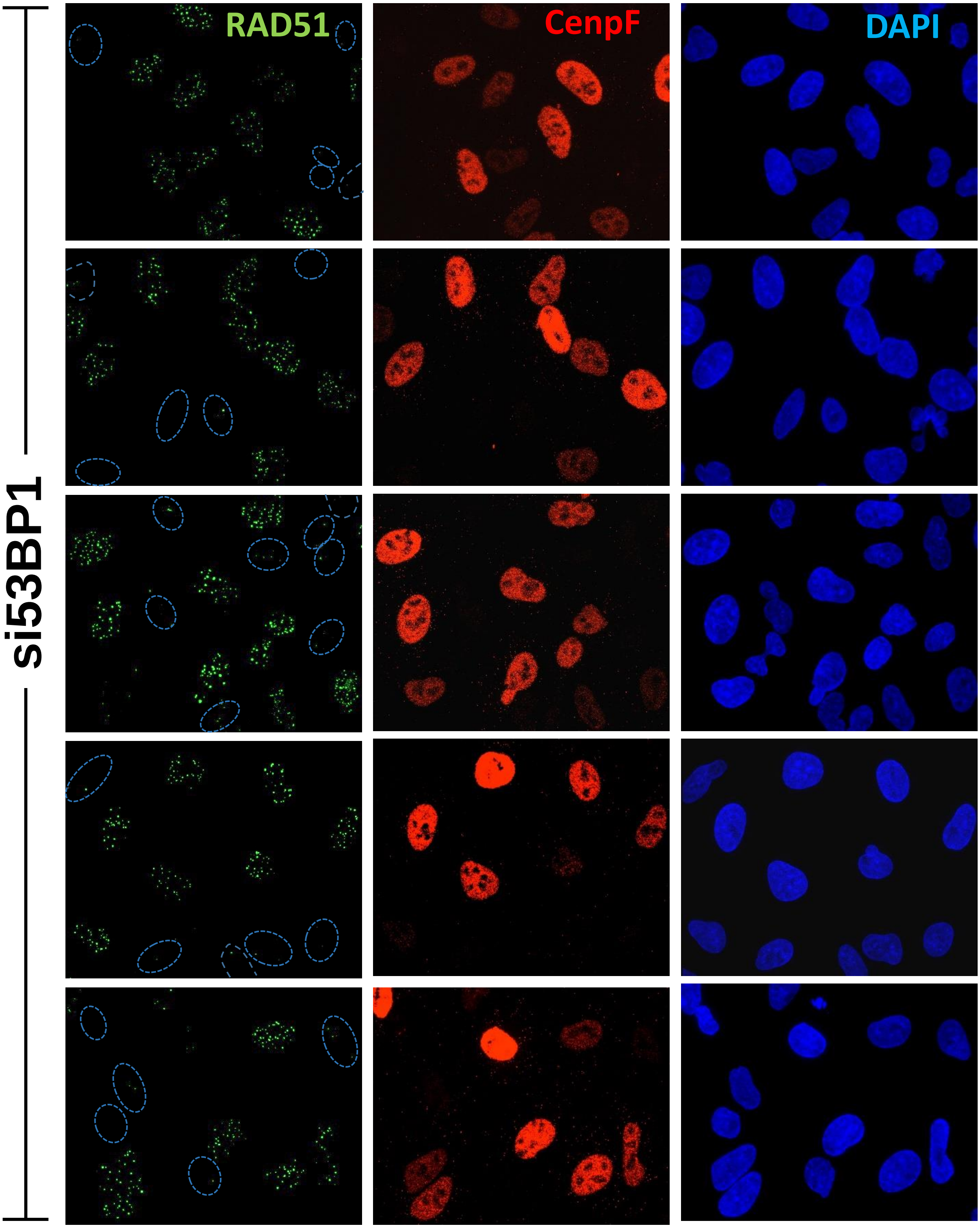

Figure S4K.

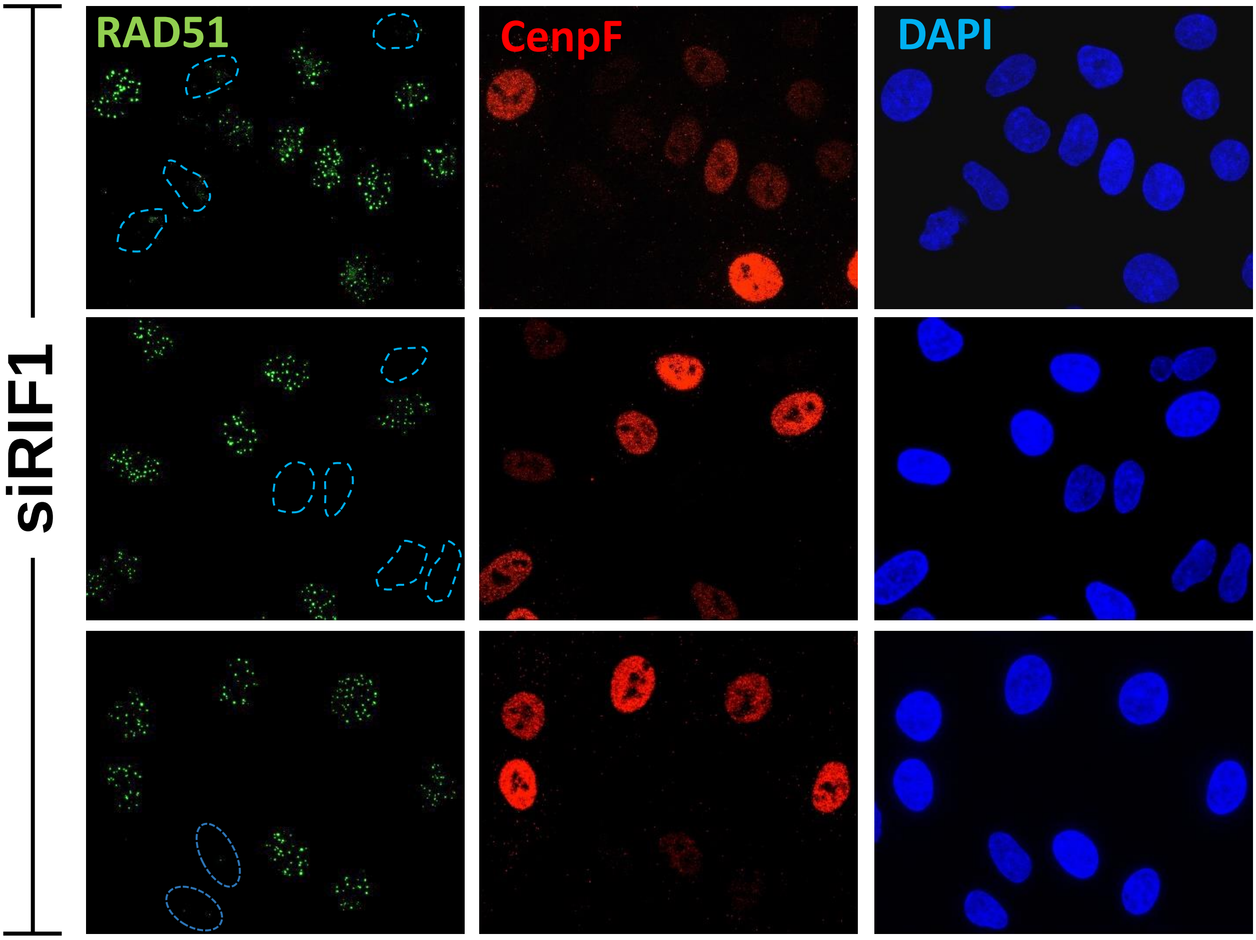

Figure S4L.

Figure S4. Representative projections of BRCA1, CtIP, RPA and RAD51 foci in G1 cells. Asynchronous A549 cells were treated with the indicated siRNA for 48h and irradiated with 2Gy. Cells were then fixed and immunostained for BRCA1 (A-C), CtIP (D-F), and RPA (G-I) after 2h or for RAD51 (J-L) after 4h post irradiation. Nuclei were counterstained with CenpF to distinguish G1 and S/G2 cells.

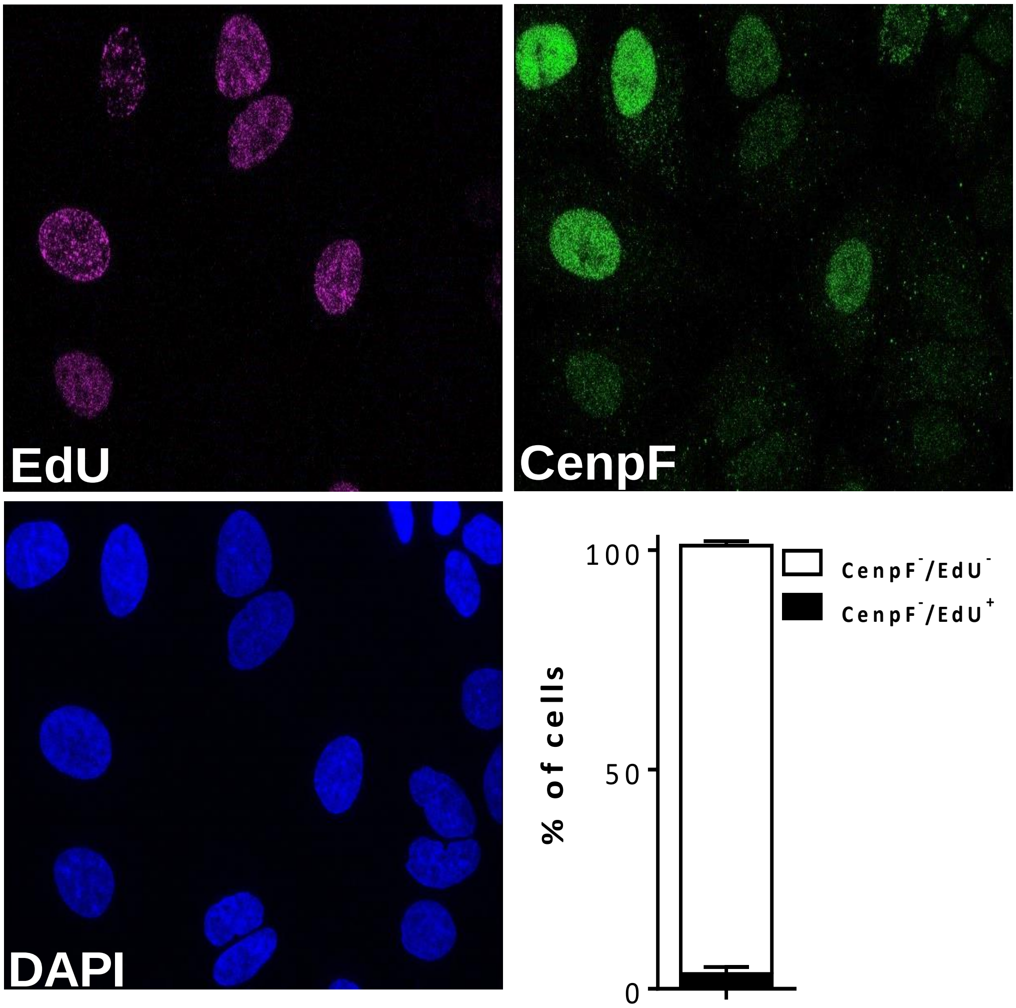

Figure S5. Double CenpF and EdU staining showed consistency in detecting G1 cells. Asynchronous A549 cells were irradiated with 2Gy and after 105 min, the dNTP analog EdU was added to the medium for 15 min and subsequently cells were fixed and stained for CenpF. Shown are the percentages of CenpF-negative cells (CenpF$^-$) which are either EdU negative (CenpF$^-$/EdU$^-$) or positive (CenpF$^-$/EdU$^+$). At least 100 nuclei were counted.
